# Heart Rate Variability and Body Motion as Digital Biomarkers of Task Workload During Military En Route Critical Care Simulations

**DOI:** 10.3390/s26113596

**Published:** 2026-06-05

**Authors:** Roger D. Dias, Sarah H. Michael, Rayan Harari, Steven Yule, Lance McGinnis, Richard J. E. Skipworth, Elizabeth Mann-Salinas, William T. Davis

**Affiliations:** 1 Department of Emergency Medicine, Harvard Medical School, Boston, MA 02115, USA; 2 Medical AI and Cognitive Engineering (MAICE) Lab, STRATUS Center for Medical Simulation, Mass General Brigham, Boston, MA 02115, USA; 3 Center for COMBAT Research, Medical Campus, University of Colorado Anschutz, Aurora, CO 80045, USA; 4 Department of Emergency Medicine, School of Medicine, University of Colorado Anschutz, Aurora, CO 80045, USA; 5 Department of Radiology, Mass General Brigham, Boston, MA 02115, USA; 6 Surgical Sabermetrics Laboratory, Usher Institute, University of Edinburgh, Scotland EH16 4TJ, UK; 7 Department of Clinical Surgery, University of Edinburgh/Royal Infirmary of Edinburgh, Scotland EH16 4TJ, UK; 8 Nursing Research Division, 59th Medical Wing Science and Technology, JBSA-Lackland, San Antonio, TX 78236, USA; 9 En Route Care Research Center, 59th Medical Wing Science and Technology, San Antonio, TX 78234, USA; 10 Department of Emergency Medicine, Brooke Army Medical Center, San Antonio, TX 78234, USA; 11 Department of Military and Emergency Medicine, Uniformed Services University, Bethesda, MD 20814, USA

**Keywords:** cognitive load, body motion, digital biomarkers, military medicine, en route critical care, medical simulations

## Abstract

Assessing task workload during military En Route Critical Care (ERCC) and other high-risk clinical operations remains challenging. This study evaluated heart rate variability (HRV) and body motion as digital biomarkers of task workload among Critical Care Air Transport (CCAT) teams during simulated ERCC missions. Seventy-two U.S. Air Force clinicians participated in 26 high-fidelity simulations. HRV and body motion were continuously captured via a chest-strap wearable sensor, and the NASA Task Load Index (NASA-TLX) was used to assess self-reported workload. Results demonstrated high feasibility (>90% HRV, 100% motion retention) with high wearable comfort ratings. Both HRV and Motion metrics showed strong construct validity, significantly differentiating resting baseline from active simulation (all *p* < 0.001, effect sizes d = 0.54–1.22 for HRV, r = 0.92–1.00 for motion). Convergent validity was selective: only baseline-adjusted root mean square of successive differences (RMSSD) demonstrated significant associations with NASA-TLX mental demand (β = −0.250, *p* = 0.005) and temporal demand (β = −0.233, *p* = 0.003). Baseline-adjusted RMSSD effectively indexes perceived workload, while Motion metrics provide complementary information about physical task demands. These findings support multimodal approaches integrating physiological reactivity, motion patterns, and self-reported ratings for comprehensive workload assessment in complex operational settings.

## 1. Introduction

Critical Care Air Transport (CCAT) teams are a critical component of the United States (U.S.) Air Force evacuation paradigm. En Route Critical Care (ERCC) missions place CCAT teams in a uniquely demanding sociotechnical system: a “portable ICU” delivered in-flight under severe operational constraints [1,2]. In addition to the clinical complexity of caring for critically ill patients, teams must function in a transport environment characterized by limited space, restricted access to patients and equipment, noise/vibration, and other flight-related stressors that can degrade monitoring, communication, and fine motor performance [3,4]. In this setting, errors can have immediate consequences, and team performance must remain reliable despite time pressure, high uncertainty, and interruptions. Importantly, simulation-based evidence in CCAT missions indicates that “task saturation” occurs frequently during crisis events and is associated with poorer teamwork and communication, deteriorating clinical performance [1]. Prior research in real clinical settings has also demonstrated the link between non-technical skills (i.e., cognitive and social) and patient outcomes [5], underscoring the operational importance of understanding and monitoring cognitive workload and its impact on clinical performance.

Task workload is a multidimensional construct describing the portion of an individual’s limited capacity required to meet task demands within a given context [6,7]. Contemporary human factors frameworks emphasize that workload reflects an interaction among task demands, operator characteristics (e.g., expertise, fatigue), and environmental constraints, and that “redlines” can be reached where performance deteriorates or becomes brittle [7]. In safety-critical domains, excessive workload is linked to decrements in attention allocation, decision quality, and error management, while insufficient workload can also impair vigilance and situation awareness [7]. Consequently, measuring task workload is central to evaluating system design, training effectiveness, and operational readiness in emergency, critical care, and other high-risk activities where clinicians must make rapid decisions under pressure [6,8,9].

Despite its importance, routine workload assessment in operational environments remains limited. Widely used approaches rely heavily on subjective ratings (e.g., NASA-TLX), which are practical and informative but typically retrospective and therefore less suited for continuous, real-time monitoring during dynamic events [7,10]. Observational and performance-based measures can add context but are resource-intensive and may be difficult to implement consistently in realistic training or operational settings [6,11]. These constraints are especially relevant for the ERCC setting, where workload may fluctuate rapidly with evolving patient physiology, equipment issues, aircraft events, and communication demands. Accordingly, there is a clear need for objective, time-sensitive measures that can index workload continuously and unobtrusively, supporting more granular evaluation of training scenarios, team processes, and emerging overload states [9,12].

Digital biomarkers—objective, quantifiable physiological or behavioral measures captured through connected digital devices—offer a promising pathway to close this gap [13]. Digital biomarkers can support continuous measurement in both real and simulated clinical environments and may be “fit-for-purpose” when appropriately verified and validated for a specific context of use [9,14]. For indexing workload, two candidate biomarkers are particularly compelling. First, heart rate variability (HRV) provides well-established indices of autonomic regulation [15,16] and has demonstrated sensitivity to detect changes in cognitive workload and acute stress, including robust effects on HRV dynamics during demanding cognitive clinical tasks [9,17]. Second, movement-derived metrics from wearable inertial sensors can capture behavioral adaptations to task demands; empirical work shows that posture and movement patterns change systematically with increasing mental workload [18], and are associated with clinical team non-technical skills [19]. Furthermore, combined heart rate and motion signals from wearables can differentiate workload conditions under certain activity contexts [20]. Combined, HRV and body-motion features represent plausible digital biomarkers that could enable unobtrusive, real-time workload assessment during ecologically valid ERCC training and future real-life military medical operations.

Building on these foundations, the present study evaluates whether wearable-derived HRV and body motion can function as digital biomarkers of task workload in a simulated military ERCC setting. More specifically, the study aimed to investigate the feasibility and gather validity evidence (known-group construct validity and convergent validity) of HRV and body Motion metrics captured via an unobtrusive wearable sensor, as digital biomarkers of task workload among military CCAT teams during ERCC simulations.

## 2. Methods

### 2.1. Study Design and Setting

**Figure 1:**
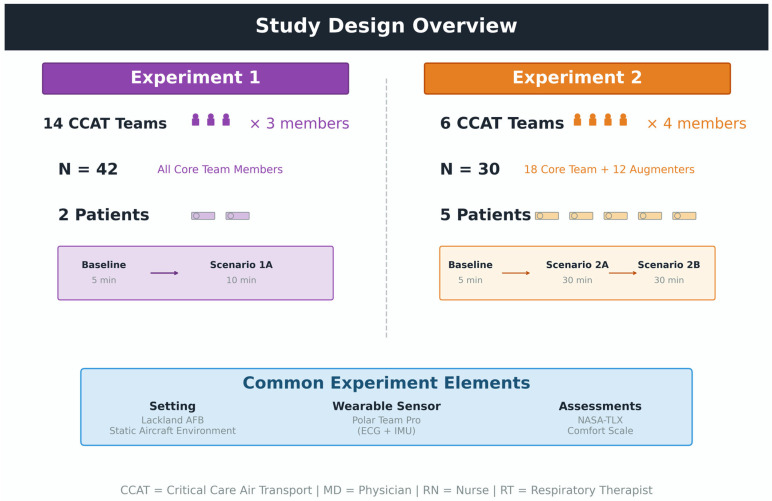
Overview of the study design encompassing experiments 1 and 2.

This was a prospective observational study with repeated measures conducted at Lackland Air Force Base in San Antonio, Texas. The study utilized a static aircraft field environment designed to replicate operational en route care conditions characteristic of U.S. Air Force Critical Care Air Transport (CCAT) missions. The high-fidelity simulations incorporated authentic en route care medical equipment and environmental conditions, including noise and lighting parameters representative of the operational CCAT environment. The research protocol received approval from the Institutional Review Board (IRB), and all participants provided written informed consent. Data were collected from two separate experiments (Figure 1) conducted sequentially as part of an exploratory research program examining CCAT team performance under different patient load conditions and team composition:

**Experiment 1** examined 3-member teams caring for 2 patients during high-fidelity scenarios. Fourteen CCAT teams, each consisting of a physician (MD), nurse (RN), and respiratory therapist (RT), completed a single 10-min simulated scenario (Scenario 1A) following a 5-min resting baseline period (seated position).

**Experiment 2** examined 4-member teams caring for 5 patients with augmented team composition. Six CCAT teams each performed two different 30-min scenarios (Scenarios 2A and 2B). Each team comprised a core team of three members plus one augmenter. Core team members participated in both scenarios, while each augmenter participated in a single scenario. Each simulation was preceded by a 5-min resting baseline period (seated position). The 5-patient scenarios differed between Scenario 2A and 2B, with simulated combat-wounded patients requiring different medical interventions (detailed scenarios available in Supplementary Materials File S1.

### 2.2. Population

Participants were military clinicians consisting of physicians, nurses, respiratory therapists, and paramedics providing critical care during aeromedical evacuation. All participants were from clinical specialties eligible for en route care, but only a portion of participants had formal CCAT training prior to enrollment. Teams were configured to model operational CCAT team composition used during military en route care missions (Physician, Nurse, and Respiratory Therapist). Demographic data were collected at the beginning of each study session, including age, military status (Active Duty, Reserve, Guard), sex, job role (Physician, Nurse, Respiratory Therapist, Paramedic), years of practice, and prior formal CCAT training (Yes/No). There was no overlap of participants between experiments 1 and 2.

### 2.3. Procedures

**Wearable Sensor Technology:** Continuous physiological and motion data were collected using the Polar Team Pro (Polar Electro Oy, Kempele, Finland) wearable sensor system, a chest-strap device integrating multiple sensors. The device includes: (1) an electrocardiogram (ECG) sensor capturing RR intervals (interbeat intervals) as event-based timestamps of successive heartbeats in milliseconds [21]; and (2) an inertial measurement unit (IMU) containing a 3-axis accelerometer, 3-axis gyroscope, and 3-axis magnetometer. Movement data were calculated using proprietary 9-degree-of-freedom sensor fusion algorithms that integrate information from all three sensor types [22]. All sensor data were output at 10 Hz for analysis. The sensors incorporated built-in memory for local storage during data collection, with subsequent cloud synchronization to the Polar Flow Portal for data download and analysis. The Polar Team Pro chest strap was placed on each participant at the beginning of the study session, following the informed consent procedure, and remained in place until completion of all simulations. Previous literature supports the validity of the Polar sensors [21,23,24].

**Subjective Workload Assessment:** A paper-version of the NASA Task Load Index (NASA-TLX) was administered immediately after each simulation completion to assess self-reported task workload [25]. The NASA-TLX consists of six dimensions rated on a 0–100 scale: Mental Demand, Physical Demand, Temporal Demand, Performance (reverse scored), Effort, and Frustration. An overall workload score was calculated as the unweighted average of the six dimensions using the raw TLX method.

**Wearable Sensor Comfort Assessment:** Self-reported comfort associated with the Polar Team Pro chest strap was assessed using a validated self-report tool [21,26]. This scale ranges from 1 to 17 and was normalized to a 1–100 scale. It is an inverse scale with lower scores indicating higher comfort levels, including six dimensions: anxiety, attachment, emotion, harm, movement, and perceived change.

**Simulation Scenarios:** In Experiment 1, scenario 1A included two simulated patients. One patient had a traumatic brain injury (TBI) requiring mechanical ventilation and intracranial pressure monitoring, and the other patient had a lower extremity fracture requiring pain control. In Experiment 2, scenario 2A included four high-fidelity patient simulators requiring mechanical ventilation for the following conditions: TBI, polytrauma from blast injury, severe burn with inhalation injury, and polytrauma complicated by sepsis. In addition, one simulated non-intubated patient was included, with a lower extremity fracture and pulmonary embolism. Scenario 2B had the same patient composition as scenario 2A, but different medical conditions and included four high-fidelity patient simulators requiring mechanical ventilation for the following conditions: TBI, polytrauma complicated by acute respiratory distress syndrome, polytrauma with temporary abdominal closure, and crush injury. The one simulated non-intubated patient had a hemothorax from a gunshot wound.

Participant posture varied across phases of the protocol. In both experiments, the 5-min resting baseline was conducted seated. In Experiment 1, participants stood continuously for the entirety of the 10-min scenario, which began with the aircraft already at altitude. In Experiment 2, the 30-min scenarios followed a scripted flight profile: participants stood during minutes 0:00–7:00 (pre-flight patient stabilization), were seated for the simulated take-off during minutes 7:00–10:00, and stood again for the remainder of the scenario (minutes 10:00–30:00, in-flight active care at altitude). During the seated take-off phase, clinicians remained actively engaged in cognitive work—planning the care of the five critically ill patients, anticipating likely clinical deteriorations, and communicating with team members about role assignments and care priorities.

Detailed descriptions of the simulation scenarios are provided in the Supplementary Materials File S1.

### 2.4. Data Processing

**Software Environment:** All data processing and analyses were performed in *Python* 3.9 programming language using the following packages: *NeuroKit2* (v0.2.7) [27] for HRV signal processing and parameter calculations, *pandas* (v2.0.3) for data manipulation and descriptive statistics, and *NumPy* (v1.24.3) for numerical computations.

**Sliding Window Analysis:** To capture dynamic physiological changes during continuous task performance, a sliding window approach was applied to both HRV and motion data. A window size of 120 s with 50% overlap (60-s steps) was used, with a start offset of −60 s to generate HRV parameters for every minute of the baseline and simulation periods. Each period was divided into overlapping 120-s windows, advancing in 60-s increments. Prior research has demonstrated that ultra-short HRV segments (approximately 1–2 min) provide valid estimates of autonomic modulation and are appropriate for task-based and dynamic operational contexts, where longer stationary windows may obscure rapid changes in workload and physiological state [16,28,29].

**Heart Rate Variability Metrics:** RR interval data in milliseconds were processed using *NeuroKit2*’s built-in functions. Peak detection and cleaning were performed by converting RR intervals to peak indices (1000 Hz sampling rate for millisecond precision). Artifact detection was performed by flagging successive RR interval differences greater than 20% as likely artifacts, following common HRV preprocessing heuristics [30,31]. Three HRV metrics were calculated:

- **RMSSD (root mean square of successive differences, ms):** A time-domain metric reflecting parasympathetic nervous system activity, calculated using *NeuroKit2*’s hrv_time() function.
- **LF/HF Ratio (unitless):** A frequency-domain metric reflecting sympathovagal balance, calculated as the ratio of Low Frequency power (0.04–0.15 Hz) to High Frequency power (0.15–0.40 Hz) using Welch’s periodogram method in *NeuroKit2*’s hrv_frequency() function.
- **Heart Rate (beats per minute—BPM):** Cardiovascular activity, calculated as the mean of instantaneous heart rates (60,000/RR interval in ms).

**Motion Metric Derivation:** The Polar Team Pro uses proprietary algorithms to derive Motion metrics from the IMU sensors [22,23]. Speed and distance are calculated using inertial dead reckoning, where accelerometer data are integrated over time to estimate velocity, and velocity is integrated to calculate distance traveled. The gyroscope provides orientation data to transform acceleration measurements from the sensor coordinate frame to the world coordinate frame, enabling the removal of gravitational acceleration (9.81 m/s^2^). Cadence is calculated through stride detection algorithms that identify rhythmic stepping patterns from accelerometer signals, detecting peaks in acceleration patterns to count steps within time windows, which are then converted to steps per minute. Seven Motion metrics were derived from the IMU sensor data:

- ***Acceleration Standard Deviation*** (Acc SD, m/s^2^): Movement variability.
- ***Maximum Acceleration*** (Acc Max, m/s^2^): Peak movement intensity.
- ***Mean Speed*** (Speed Mean, km/h): Average movement velocity.
- ***Speed Standard Deviation*** (Speed SD, km/h): Speed variability.
- ***Maximum Speed*** (Speed Max, km/h): Peak velocity.
- ***Distance*** (m, meters): Total distance traveled per 120-s window, calculated from cumulative displacement.
- ***Active Cadence Percentage*** (Cadence Active, %): Percentage of time within the window during which stepping activity was detected, with built-in noise filtering at 32.0 steps/min.

**Data Quality Control:** Data quality control was implemented as a multi-level filtering pipeline applied before aggregation and analysis as described below:

***HRV Data Quality Control* involved three levels:**

- ***Level 1*** *(Window Processing Failures)* identified windows with insufficient or physiologically invalid RR interval data based on: (1) unsuccessful RR interval extraction due to signal loss, excessive noise, or file corruption; (2) >10% physiologically implausible RR intervals (outside 300–2000 ms range, corresponding to heart rates outside 30–200 BPM); (3) insufficient RR intervals after outlier removal (<100 beats for combined RMSSD and LF/HF analysis, or <50 beats for RMSSD-only analysis); or (4) invalid computational results (NaN or infinite values). Windows with ≤10% outliers had outliers removed and proceeded to HRV calculation; windows with >10% outliers were excluded.
- ***Level 2*** *(Poor Quality Windows)* excluded windows that passed Level 1 processing but contained excessive artifacts. Quality scores were calculated as the sum of outlier percentage (≤10% by definition) and artifact percentage (successive RR differences >20%). Windows with >10% total quality issues were classified as “Poor” and excluded.
- ***Level 3*** *(Subject-Period Exclusions)* excluded subject-period combinations where >50% of successfully processed windows were rated “Poor” quality, applied separately for each subject × period combination (Baseline, Scenario 1A, Scenario 2A, and Scenario 2B).

***Motion Data Quality Control*** applied a single criterion: data completeness <50% of expected samples per window, at a 10 Hz sampling rate, led to window exclusion.

**Data Aggregation:** A two-step median aggregation approach was used. In Step 1 (Within-Period Window Aggregation), all valid windows for each subject-period combination were aggregated using the median to minimize the influence of outlier windows while preserving individual physiological responses. This produced one value per subject-period for each metric. In Step 2 (Across-Subjects Statistics), the within-subject aggregated values from Step 1 were used to calculate group-level statistics, including median, Q1 (25th percentile), and Q3 (75th percentile). For Experiment 2, the two scenarios (Scenario 2A and Scenario 2B) were maintained as separate observations in all HRV and motion analyses. This approach maximized statistical power by including all available scenario observations while properly accounting for within-subject correlation through random effects modeling (i.e., subject-level random intercepts in all linear mixed-effects models). NASA-TLX responses were similarly analyzed at the scenario level, with each scenario contributing independently to the analysis while the statistical models controlled for repeated observations from the same subjects.

### 2.5. Statistical Analysis

**Construct Validity (Baseline vs. Simulation Comparison):** To evaluate the known-group construct validity [32,33], we examined whether HRV and Motion metrics changed as expected from resting baseline to simulation scenarios, combining data from both studies.

***HRV Metrics:*** Linear mixed-effects models (LMM) were used to compare baseline versus simulation conditions across all participants, accounting for within-subject correlation through random intercepts. Two models were fit for each HRV metric:

- ***Unadjusted Model:*** Outcome ~ Condition + (1|Subject)—tests raw condition effect.
- ***Adjusted Model:*** Outcome ~ Condition + Experiment + Age + CCAT_Training + Sex + (1|Subject)—controls for covariates to isolate pure condition effects.

The random effect (1|Subject) accounts for within-subject correlation, including repeated scenarios for Experiment 2 core team members. Covariates were selected a priori based on the previous literature [34,35] on the effect of age, sex, and expertise (prior CCAT Training) on HRV, and study design considerations (Experiment 1 vs. Experiment 2). Multicollinearity among covariates was assessed using Variance Inflation Factors (VIF), with all values <5.0 indicating acceptable independence: Experiment (VIF = 2.34), Age (VIF = 4.99), prior CCAT training (VIF = 2.99), and Sex (VIF = 2.42). Effect sizes were calculated as Cohen’s d.

***Motion Metrics:*** Wilcoxon signed-rank tests were used for within-subject paired comparisons because distributions were severely non-normal, with many zero values at baseline under the resting condition. Effect sizes were calculated as r = Z/√N.

**Convergent Validity (Digital Biomarkers and Self-Report Workload):** To evaluate convergent validity, we examined correlations between digital biomarkers and NASA-TLX ratings using scenario-level data. Two complementary approaches were employed: (1) Spearman’s rank correlation (ρ) for descriptive effect size estimates, and (2) linear mixed-effects models (LMM) with robust standardization (median and IQR) for valid statistical inference accounting for within-subject correlation from Experiment 2 Core Team members completing multiple scenarios. The LMM specification was: NASA_TLX_z ~ Physiological_z + (1|Subject).

For HRV metrics, a sensitivity analysis accounted for individual baseline differences using the model: NASA_TLX_z ~ Simulation_HRV_z + Baseline_HRV_z + (1|Subject), where *β_simulation* tests HRV reactivity (change from baseline) and *β_baseline* captures individual baseline (trait) differences. This baseline-adjusted approach addresses the well-established finding that HRV reactivity may be more relevant in capturing acute workload than absolute values [36,37,38].

To assess independence of digital biomarker modalities, correlations between HRV and Motion metrics during simulation were examined using: HRV_z ~ Motion_z + (1|Subject). This analysis tested whether autonomic nervous system activity (HRV) and physical activity (motion) represent independent or overlapping indicators of task workload.

**Exploratory Subgroup Analyses:** Five exploratory subgroup analyses examined whether digital biomarker responses and subjective workload differed by study design, prior CCAT training, job role, and demographic characteristics.

***Experiment 1 vs. Experiment 2 Comparison*:** To investigate the potential impact of higher patient volume and different team composition, we compared Experiment 1 (2-patient/3-member teams) versus Experiment 2 (5-patient/4-member teams). For HRV metrics, interaction analysis tested whether baseline-to-simulation changes differed between studies using: Outcome ~ Condition × Experiment + (1|Subject), where the interaction term tests differential physiological response. For NASA-TLX and Motion metrics, between-study comparisons used: Outcome ~ Experiment + (1|Subject). Unadjusted models were used to estimate total experiment differences, as team composition, prior CCAT training, and other design features are inherent to each experiment rather than confounders to control.

***Demographics and Job Role Subgroup Analyses*:** To examine whether digital biomarker responses varied by clinician characteristics, subgroup analyses tested for differential effects by prior CCAT training (Yes vs. No), job role (Nurse, Physician, Respiratory Therapist, excluding 1 Paramedic), age (continuous variable, years), sex (Male vs. Female), and years of practice (continuous variable, years).

For HRV reactivity in categorical subgroups, interaction models tested differential baseline-to-simulation changes: Outcome ~ Condition × Group + Experiment + (1|Subject), where the interaction term tests whether the group showed different physiological responses, with Experiment included as a covariate to control for design differences between Experiment 1 and Experiment 2.

For continuous predictors (Age, Years of Practice), HRV reactivity analyses used: Outcome ~ Condition × Continuous_Predictor + Experiment + (1|Subject), where the interaction term tests whether the continuous variable modulates physiological response.

For Motion metrics and NASA-TLX in categorical subgroups, between-group comparisons during simulation used: Outcome ~ Group + Experiment + (1|Subject).

For continuous predictors (Age, Years of Practice), motion and NASA-TLX analyses used: Outcome ~ Continuous_Predictor + Experiment + (1|Subject). Job role analyses used pairwise comparisons (Nurse vs. Physician, Nurse vs. RT, Physician vs. RT).

Statistical significance was set at α = 0.05. All analyses were performed using *Python 3.9* with *statsmodels* and *scipy* packages.

**Power Analysis:** Sample size was determined a priori based on effect sizes reported in a pilot study examining HRV-based cognitive load measurement during simulation-based trauma team training [9]. This study reported LF/HF ratio differences between simulation scenarios (active trauma care) and debriefing periods (seated discussion) with effect sizes (Cohen’s dz) ranging from 0.44 to 0.67 (mean dz = 0.56). Using the average effect size from scenario comparisons (dz = 0.56), a minimum of 36 participants was required to achieve 90% power at alpha = 0.05 (two-tailed paired *t*-test). The current study analyzed 72 participants, exceeding the minimum requirement.

A *post hoc* power analysis was conducted using observed effect sizes from the current study, confirming adequate statistical power for all primary analyses. Construct validity analyses comparing baseline and simulation phases achieved >99% power for HRV metrics (Cohen’s d = 0.56–1.22, *n* = 69) and 100% power for Motion metrics (r = 0.92–1.00, *n* = 72). Convergent validity analyses examining associations between RMSSD and NASA-TLX subscales achieved 94–98% power (standardized beta = −0.23 to −0.27, *n* = 56). Subgroup analyses comparing physiological reactivity across experiment cohorts, training levels, and job roles achieved 72–97% power (Cohen’s d = 0.44–0.67, *n* ~ 35 per group). Note that exploratory subgroup comparisons for experiment cohort differences in HR (73%), training level effects (72%) were underpowered.

**Posture-stratified sensitivity analysis:** To address the potential confounding effect of postural change between the seated resting baseline and the predominantly standing simulation phases, we conducted a posture-stratified sensitivity analysis leveraging the scripted seated simulated take-off period embedded in the Experiment 2 scenarios (minutes 7:00–10:00). Phase-level HRV metrics (RMSSD, LF/HF, mean HR) were computed natively from the raw R-R intervals over the full duration of each phase (seated resting baseline, standing pre-takeoff, seated take-off, standing active care), with signal-processing and quality-control parameters identical to the main pipeline. Four pre-specified contrasts comparing the four phases pairwise—two posture-matched (seated → seated and standing → standing) and two posture-switching—were fitted using linear mixed-effects models with subject random intercepts, both unadjusted and adjusted for age, sex, and prior CCAT training. Full methods and results are reported in the Supplementary Materials File S3.

## 3. Results

### 3.1. Participant Enrollment

Seventy-two military clinicians from the U.S Air Force were enrolled across both studies. Experiment 1 enrolled 45 unique participants (15 teams of three members), and Experiment 2 enrolled 30 unique participants (18 core team members and 12 augmenters—six teams of four members). One team of three participants was excluded from Experiment 1 due to overheated equipment preventing scenario completion and data capture.

### 3.2. Participant Demographics

Participant demographic characteristics are presented in Table 1. The overall cohort (*n* = 72) had a median age of 33.0 [28.0, 38.0] years and a median of 4.0 [3.0, 7.0] years of clinical practice. The sample was balanced by sex (51.4% male, 48.6% female) and consisted primarily of Active-Duty military personnel (75.0%), with the remainder from Guard (15.3%) and Reserve (9.7%) components. Job roles included nurses (47.2%), physicians (27.8%), respiratory therapists (23.6%), and one paramedic (1.4%). Forty-one participants (56.9%) had no prior formal CCAT training.

Experiment 1 participants (*n* = 42) were predominantly Active-Duty personnel (95.2%) with a median age of 31.0 [28.2, 36.8] years and 4.0 [3.0, 5.0] years of practice experience. The majority (71.4%) had no prior CCAT training. Experiment 2 participants (*n* = 30) had greater representation from Guard (33.3%) and Reserve (20.0%) components, were older (median age 35.5 [27.2, 42.0] years), had slightly more practice experience (4.5 [3.0, 13.8] years), and most (63.3%) had prior CCAT training.

### 3.3. Feasibility Findings

**Figure 2:**
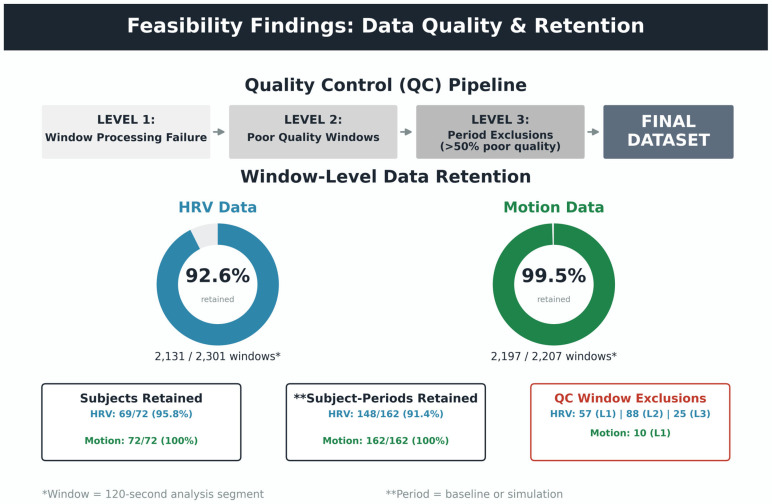
Summary of feasibility assessment findings related to data quality and retention.

All 72 analyzed participants completed the study protocol. Table 2 shows data retention across HRV and Motion metrics following quality control procedures, further illustrated in Figure 2.

**HRV Data Quality:** A three-level quality control pipeline resulted in 92.6% window retention (2131 of 2301 windows), 95.8% subject retention (69 of 72 subjects), and 91.4% subject-period retention (148 of 162 subject-periods). Level 1 excluded 57 windows (2.5%) due to processing failures, including signal loss, excessive noise, or physiologically implausible RR intervals (>10% outside the 300–2000 ms range). Level 2 excluded 88 windows (3.9% of Level 1 successful windows) with >10% combined outlier and artifact percentages. Level 3 excluded six subject-period combinations where >50% of successfully processed windows were rated “Poor” quality.

**Motion Data Quality:** Motion data demonstrated excellent quality with 99.5% window retention (2197 of 2207 windows), 100% subject retention, and 100% subject-period retention. Only 10 windows (0.5%) failed quality control due to <50% data completeness.

**Figure 3:**
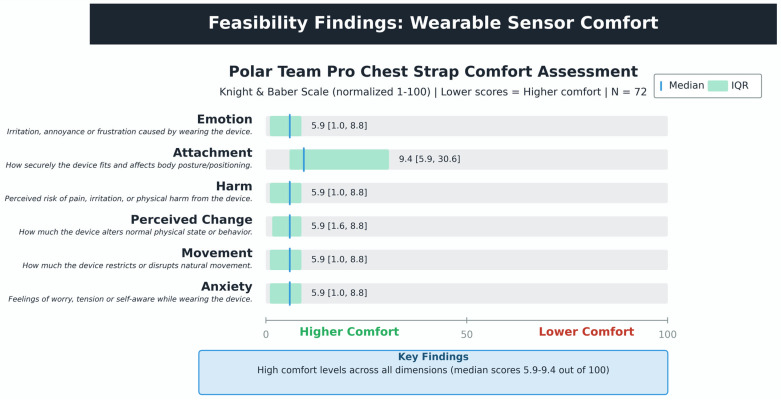
Summary of feasibility assessment findings related to wearable sensor comfort.

**Wearable Sensor Comfort:** Participants (*n* = 72) reported high comfort levels with the Polar Team Pro chest strap. The median comfort scores for individual dimensions were emotion 5.9 [1.0, 8.8], attachment 9.4 [5.9, 30.6], harm 5.9 [1.0, 8.8], perceived change 5.9 [1.6, 8.8], movement 5.9 [1.0, 8.8], and anxiety 5.9 [1.0, 8.8], as illustrated in Figure 3.

### 3.4. Construct Validity: Baseline vs. Simulation

To evaluate known-group construct validity, we examined whether digital biomarkers changed as expected during high-workload simulation scenarios compared to a resting baseline condition, combining data from both studies. Following quality control, HRV analysis included 69 subjects with 148 observations (subject-periods), and motion analysis included all 72 subjects with 162 observations. Adjusted models included Experiment (Experiment 1 vs. Experiment 2) as a covariate to account for differences in patient volume (two vs. five patients) and team composition (three vs. four team members), and prior CCAT training to account for differences in training levels between studies.

**Figure 4:**
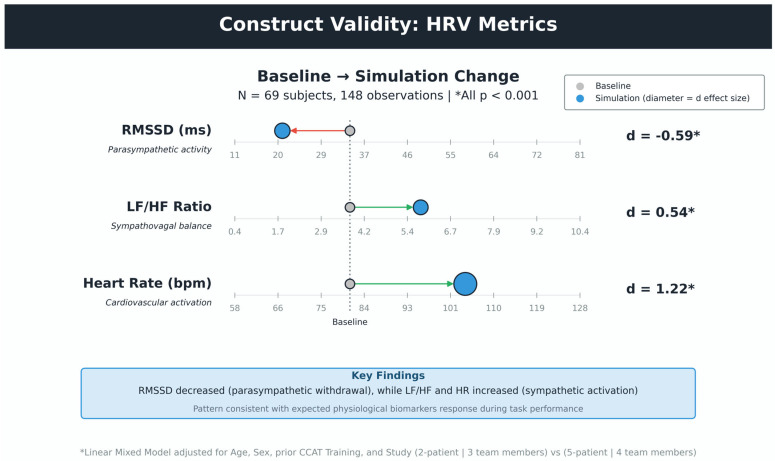
Key findings on the construct validity of HRV metrics.

**HRV Metrics:** All three HRV metrics demonstrated highly significant changes from baseline to simulation (Table 3), with effect sizes ranging from moderate to very large. Key findings are summarized in Figure 4.

***RMSSD*** decreased significantly from baseline (median 34.4 [19.9, 56.3] ms) to simulation (20.7 [12.7, 29.1] ms), representing a 39.7% reduction (unadjusted: β = −16.32 ms, 95% CI [−22.39, −10.25], *p* < 0.001, **d = −0.58**). After adjusting for Experiment 1/Experiment 2, Age, prior CCAT training, and Sex, the effect remained essentially unchanged (adjusted: β = −16.98 ms, *p* < 0.001, **d = −0.59**).

***LF/HF Ratio*** increased significantly from baseline (median 3.78 [2.27, 6.10]) to simulation (5.83 [4.41, 8.05]), representing a 54.2% increase (unadjusted: β = 1.85, 95% CI [1.12, 2.58], *p* < 0.001, **d = 0.56**). The adjusted model yielded nearly identical results (β = 1.81, *p* < 0.001, **d = 0.54**).

***Heart Rate*** showed the strongest effect, increasing significantly from baseline (median 80.9 [70.4, 92.6] bpm) to simulation (104.3 [91.6, 116.1] bpm), representing a 29.0% increase (unadjusted: β = 24.32 bpm, 95% CI [21.17, 27.48], *p* < 0.001, **d = 1.22**). The adjusted model confirmed these findings (β = 24.89 bpm, *p* < 0.001, **d = 1.22**).

**Motion Metrics:** All seven Motion metrics showed very large increases from baseline to simulation (all *p* < 0.001, **effect sizes r = 0.92–1.00**) (Table 4). Baseline values were near zero for all metrics (median 0.00 for six of seven metrics).

### 3.5. Convergent Validity: Digital Biomarkers and Subjective Workload

To evaluate convergent validity, we examined correlations between digital biomarkers and subjective workload ratings from the NASA Task Load Index (NASA-TLX), using scenario-level data from the simulation periods. After merging with quality-controlled physiological data, the convergent validity analysis included 78 paired scenarios from 61 subjects for both HRV–Motion and HRV—NASA-TLX correlations. For Motion–NASA-TLX correlations, 90 paired scenarios from 72 subjects were included.

**Unadjusted Correlations:** Absolute physiological values showed minimal convergent validity with subjective workload measures. *HRV metrics* (*RMSSD*, *LF*/*HF Ratio*, *Heart Rate*) demonstrated near-zero correlations with all NASA-TLX dimensions (all |ρ| < 0.22, |β| < 0.14, all *p* > 0.05). *Motion metrics* showed similarly weak associations with NASA-TLX ratings, with no correlations reaching statistical significance (all |ρ| < 0.21, |β| < 0.19, all *p* > 0.05). Full results are reported in Supplementary Materials File S2.

**Baseline-Adjusted HRV Analysis:** A sensitivity analysis accounting for individual baseline differences revealed two distinct patterns in how RMSSD relates to subjective workload (Table 5). This analysis required subjects with valid baseline HRV, simulation HRV, and NASA-TLX data, yielding a sample of 56 subjects with 71 scenario observations.

First, **baseline-to-simulation RMSSD reactivity** (parasympathetic withdrawal during simulation) showed significant negative associations with subjective workload across multiple NASA-TLX dimensions (*n* = 71). After controlling for baseline RMSSD, lower RMSSD values during simulation were associated with higher perceived workload for **Overall Workload** (β = −0.269, 95% CI [−0.471, −0.067], *p* = 0.009), **Mental Demand** (β = −0.250, 95% CI [−0.424, −0.075], *p* = 0.005), and **Temporal Demand** (β = −0.233, 95% CI [−0.385, −0.080], *p* = 0.003).

On the other hand, **baseline (trait) RMSSD** showed significant positive associations with perceived workload during simulations (*n* = 71). Individuals with **higher resting RMSSD** reported higher **Overall Workload** (β = +0.523, *p* = 0.004), **Mental Demand** (β = +0.483, *p* = 0.002), and **Temporal Demand** (β = +0.434, *p* = 0.002) during simulation scenarios. Neither LF/HF Ratio nor Heart Rate demonstrated significant associations with subjective workload even after baseline-adjusted analyses (all *p* > 0.05). Full results are reported in Supplementary Materials File S2.

**Relationships Between HRV and Motion Metrics:** To assess whether HRV and Motion represent independent or overlapping indicators of workload, we examined correlations between these two physiological modalities during simulation (Table 6). RMSSD showed minimal associations with Motion metrics (*n* = 90), with only one weakly significant correlation (Speed Max: ρ = −0.243, β = −0.178, *p* = 0.019) and six non-significant correlations (all *p* > 0.05). In contrast, Heart Rate demonstrated moderate positive correlations with most Motion metrics (5 of 7 significant, ρ = 0.228–0.312, β = 0.191–0.268, all *p* < 0.01). LF/HF Ratio showed no significant correlations with any Motion metric (all *p* > 0.05).

### 3.6. Exploratory Subgroup Analysis

**Figure 5:**
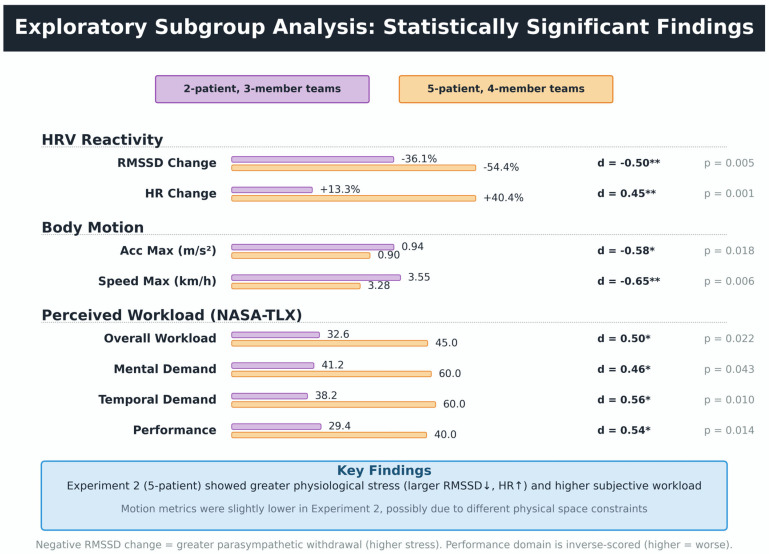
Key findings on exploratory subgroup analysis by experiment type. * *p* < 0.05, ** *p* < 0.01.

**Experiment 1 vs. Experiment 2 Conditions:** An exploratory subgroup analysis examined whether higher patient volume (five vs. two patients) combined with different team compositions (four-member vs. three-member teams), in Experiment 2, compared to Experiment 1, was associated with differential digital biomarker responses and subjective workload. Detailed comparison results are reported in Table 7, and a summary of key findings is displayed in Figure 5.

**Demographics, Training, and Experience:** To examine whether digital biomarker responses varied by clinician characteristics, exploratory subgroup analyses tested for differential effects by prior CCAT training, years of practice, job role, and demographic (sex and age) characteristics. All models included Experiment as a covariate to control for differences in patient volume and team composition between Experiment 1 and Experiment 2.

***Prior CCAT Training:*** Trained clinicians showed significantly larger Heart Rate increases (greater cardiovascular activation) during simulations compared to untrained clinicians (interaction β = 8.96 bpm, 95% CI [3.36, 14.56], *p* = 0.002, d = 0.44), with trained individuals increasing from 76.4 to 101.0 bpm (32.3% increase) versus untrained increasing from 88.5 to 105.5 bpm (19.2% increase). No significant differences were observed for RMSSD reactivity (*p* = 0.085), LF/HF reactivity (*p* = 0.530), subjective workload ratings (all *p* > 0.07), or Motion metrics (all *p* > 0.29).

***Job Role:*** Job role analyses revealed a hierarchical pattern of cardiovascular responses (Respiratory Therapists > Physicians > Nurses). Respiratory Therapists demonstrated significantly larger Heart Rate increases compared to Physicians (interaction β = 13.74 bpm, 95% CI [5.54, 21.94], *p* = 0.001, d = 0.67) and showed significantly larger RMSSD decreases (greater parasympathetic withdrawal) compared to Physicians (interaction β = −12.69 ms, 95% CI [−23.06, −2.32], *p* = 0.017, d = −0.63). Physicians demonstrated significantly larger Heart Rate increases compared to Nurses (interaction β = −6.79 bpm, 95% CI [−12.41, −1.17], *p* = 0.018, d = −0.35). RTs reported significantly higher Physical Demand ratings than Nurses (β = 10.41, *p* = 0.041, d = 0.55) and demonstrated significantly higher Cadence Active Time than Nurses (β = 1.93, *p* = 0.024, d = 0.64), partially explaining their larger cardiovascular responses (increased HR).

***Sex:*** No significant differences were observed in HRV reactivity between males and females (RMSSD: *p* = 0.075, Heart Rate: *p* = 0.087). During simulation performance, females demonstrated significantly higher maximum speed (β = −0.281 km/h, 95% CI [−0.546, −0.016], *p* = 0.038, d = −0.48). No significant differences were observed in other Motion metrics or NASA-TLX dimensions.

***Age:*** Age showed no significant associations with HRV reactivity: RMSSD (interaction β = 0.246 ms per year, 95% CI [−0.473, 0.966], *p* = 0.502, d = 0.009), LF/HF Ratio (β = −0.077 per year, 95% CI [−0.168, 0.014], *p* = 0.096, d = −0.023), or Heart Rate (β = −0.098 bpm per year, 95% CI [−0.500, 0.304], *p* = 0.633, d = −0.005). This indicates that baseline-to-simulation HRV changes did not differ significantly across the age range studied. Similarly, age was not significantly associated with Motion metrics (all *p* > 0.70) or NASA-TLX dimensions (all *p* > 0.18). This indicates that movement patterns and perceived workload did not differ significantly across the age range studied.

***Years of Practice:*** Years of practice showed no significant associations with HRV reactivity: RMSSD (interaction β = 0.295 ms per year, 95% CI [−0.752, 1.343], *p* = 0.581, d = 0.010), LF/HF Ratio (β = −0.060 per year, 95% CI [−0.191, 0.072], *p* = 0.375, d = −0.018), or Heart Rate (β = −0.079 bpm per year, 95% CI [−0.655, 0.498], *p* = 0.789, d = −0.004). This indicates that baseline-to-simulation HRV changes did not differ significantly across the range of clinical experience studied. Motion metrics (all *p* > 0.16) and NASA-TLX (all *p* > 0.05) ratings showed no significant associations with years of practice.

Full results for all subgroup analyses are reported in Supplementary Materials File S2. An overall summary of the study findings supporting multimodal assessment of task workload is shown in Table 8.

**Posture-stratified sensitivity analysis (Supplementary Materials File S3).** A posture-matched (seated → seated) contrast comparing the seated resting baseline against the seated simulated take-off phase in Experiment 2 (*n* = 28) confirmed significant HRV reactivity in the absence of postural change: RMSSD decreased by 22.9 ms (d = −0.80, *p* < 0.001), LF/HF ratio increased by 1.81 (d = +0.57, *p* = 0.014), and heart rate increased by 20.2 bpm (d = +1.43, *p* < 0.001).

## 4. Discussion

This study evaluated whether wearable-derived heart rate variability (HRV) and body motion could function as digital biomarkers of task workload during high-fidelity military En Route Critical Care (ERCC) simulations in an aircraft environment. Three principal findings emerged. First, wearable data collection was operationally feasible and well tolerated in a demanding, team-based simulation environment. Second, both HRV and motion robustly differentiated resting baseline from active simulation, providing strong known-groups construct validity. Third, convergent validity with subjective workload was selective: only baseline-adjusted RMSSD demonstrated meaningful associations with NASA-TLX, underscoring the importance of modeling physiological *reactivity* rather than absolute values when assessing acute workload.

From a feasibility perspective, the high retention of analyzable HRV and motion data, combined with favorable comfort ratings extends prior work [9,22], demonstrating that wearable physiological sensing can be deployed in applied settings with minimal participant burden. This finding is nontrivial in the ERCC context, where traditional workload assessment approaches, such as expert observation, self-report surveys, secondary-task techniques, or detailed behavioral coding, are resource-intensive and difficult to implement consistently. Consistent with long-standing calls in the human factors and cognitive engineering literature [7,39], the present results show that unobtrusive sensing can support scalable, continuous workload assessment in complex, operationally constrained environments.

The observed baseline-to-simulation changes in HRV provide strong evidence of known-groups construct validity and align with prior psychophysiological research on workload and stress in high-risk clinical settings. Decreases in RMSSD alongside increases in LF/HF ratio and heart rate are consistent with a shift in sympathovagal balance toward sympathetic predominance during the active simulation phase, achieved through both increased sympathetic activation and concurrent parasympathetic (vagal) withdrawal—a pattern well established under conditions of elevated cognitive demand, time pressure, and acute stress [16,36,38,40,41]. RMSSD, as a vagally mediated time-domain index, most directly captures the parasympathetic component of this shift, whereas LF/HF reflects the balance between the two limbs of the autonomic nervous system rather than pure sympathetic activation [42]. Heart rate, which is influenced by both sympathetic activation and vagal withdrawal, rose accordingly. This study adds to the existing literature by providing evidence that these patterns generalize beyond controlled laboratory paradigms to a realistic, team-based, highly dynamic clinical environment characterized by noise, spatial constraint, and concurrent physical activity.

Motion metrics also demonstrated clear known-groups validity, with large increases from baseline to simulation. This finding is consistent with prior ergonomic and behavioral workload research showing that observable activity increases with task engagement and complexity, particularly in physically embodied work domains [7,20,42]. At the same time, the magnitude of these motion effects highlights a critical interpretive issue emphasized in earlier human factors work: physical activity is an inherent component of many operational tasks and does not map directly onto perceived cognitive workload [7,20,43]. Research in operating room settings has demonstrated that clinician movement patterns are driven primarily by patient care tasks, equipment management, role responsibilities, and spatial layout rather than mental demand per se [44]. In the ERCC environment specifically, teams must coordinate care for multiple critically ill patients while managing portable ventilators, monitors, and infusion pumps within the confined space of an aircraft cabin [1,4,44]. Consequently, increased movement during ERCC simulations likely reflects these task-coordination demands and spatial constraints as much as mental or temporal demand. This dissociation between motion and subjective workload underscores the importance of multimodal assessment approaches that can differentiate physical task demands from cognitive load.

Convergent validity analyses refined these interpretations. Among all candidate metrics, only RMSSD, when modeled as a baseline-adjusted change, demonstrated meaningful associations with NASA-TLX dimensions. This pattern is consistent with prior recommendations that time-domain HRV measures, particularly RMSSD, are better suited to capturing short-term autonomic responses to cognitive and emotional demands than frequency-domain indices (e.g., LF-HF ratio) [42] or heart rate alone [45,46]. The present findings extend this work by demonstrating that RMSSD is most informative when treated as a within-person reactivity measure, rather than as an absolute value, in heterogeneous clinical teams.

The lack of convergent validity between Motion metrics and subjective workload, and the limited convergence observed for heart rate, further align with prior cautions in the workload literature [7,47]. Subjective workload ratings represent a retrospective, integrative appraisal of task demands, whereas heart rate and movement respond continuously to both cognitive and physical influences. In mobile, highly dynamic team-based care, such as in ERCC, heart rate is particularly sensitive to posture changes and physical activity, a limitation emphasized in foundational HRV standards [41] and subsequent methodological guidance.

Taken together, these findings support contemporary views of workload as a multicomponent construct that cannot be adequately captured by any single metric [47]. Prior theoretical and empirical work in cognitive engineering has argued for multimodal workload assessment approaches that integrate physiological, behavioral, and subjective measures [7,48]. The present study provides concrete empirical evidence for this position in the ERCC context. HRV reactivity, particularly RMSSD, appears well suited to indexing acute internal workload responses; Motion metrics provide essential contextual information about task execution and physical demand; and subjective ratings capture participants’ integrated experience of task difficulty. Partial convergence and systematic dissociation across modalities should therefore be interpreted as informative features of workload (i.e., additional components or different constructs) in complex sociotechnical systems, rather than as measurement failure.

The practical utility of the findings extends along three lines. First, unobtrusive wearable monitoring during high-fidelity CCAT training enables individualized post-scenario debriefing grounded in objective physiological reactivity rather than self-report alone, supporting targeted feedback on workload management and team coordination. Second, the demonstrated sensitivity of baseline-adjusted RMSSD to mental and temporal demand offers a foundation for near-real-time detection of emerging task-saturation states during training and, with further validation, during real-life en route care operations, where early identification of high-workload conditions could trigger team-level mitigation strategies. Third, the multimodal framework—combining HRV reactivity, motion patterns, and self-reported workload—provides an objective benchmark against which to evaluate training interventions, team composition changes, equipment redesigns, and emerging adaptive technologies for the en route care environment.

Several limitations define the boundary conditions of these conclusions. The simulations were conducted in a static aircraft environment and did not include in-flight vibration or sustained operational stressors, which may further influence physiological responses. Baseline periods were brief and seated, while simulation involved role-dependent movement, potentially contributing to some cardiovascular differences. Motion metrics were derived from proprietary algorithms, limiting transparency regarding feature derivation. While subjective workload was measured using a validated instrument, the study did not directly link physiological reactivity to objective performance or patient-care outcomes—a limitation shared by much of the existing workload biomarker literature.

Posture is a well-recognized modulator of HRV, and the contrast between the seated resting baseline and the predominantly standing simulation phase introduces a potential postural confounder for the construct-validity comparison. We addressed this directly by leveraging the seated simulated take-off phase embedded in the Experiment 2 scenarios to perform a posture-matched sensitivity analysis (Supplementary Materials File S3), in which all three HRV metrics shifted significantly in the expected direction with no change in posture. The present design does not allow a complete partitioning of workload, posture, and physical-activity components from a single contrast; a future protocol incorporating a standing-rest baseline arm would enable a fully quantitative decomposition and is an important direction for subsequent work.

Lastly, participants were aware that they were taking part in a simulation rather than responding to a real operational event, a factor that may attenuate the magnitude of stress-related physiological reactivity and self-reported cognitive demand relative to real-world en route care. While substantial effort was directed at optimizing the realism of the simulated scenario—including a static aircraft field environment, authentic en route care medical equipment, operationally representative noise and lighting conditions, and high-fidelity patient simulators—additional research is needed to establish the generalizability of these findings to real-life operational medicine encounters.

Future research should build on these findings by incorporating explicit workload manipulations, time-synchronized task annotations, and longitudinal modeling of within-scenario workload trajectories. Prior work suggests that workload dynamics, not just peak levels, are critical for understanding performance degradation and task saturation. Extending multimodal workload assessment to real-life operational environments and linking physiological reactivity to objective performance and team coordination measures will be essential steps toward operational deployment.

In summary, this study demonstrates that wearable sensing of HRV and motion is feasible in ERCC simulation and provides strong known-groups validity evidence. Baseline-adjusted RMSSD emerged as the most informative physiological indicator of perceived workload reactivity, while Motion metrics and HR offered valuable contextual insight into task-related cardiovascular demands. By explicitly situating these findings within prior workload and psychophysiology research, the study advances multimodal, objective workload measurement in complex, high-risk healthcare delivery systems.

**Disclaimer:** The views expressed are those of the authors and do not reflect the official views or policy of the Department of Defense or its Components,. or the Henry M. Jackson Foundation for the Advancement of Military Medicine, Inc. The study referenced was conducted under a protocol reviewed and approved by the San Antonio Institutional Review Board and in accordance with the approved protocol. The voluntary, fully informed consent of the subjects used in this research was obtained as required by 32 CFR 219 and DODI 3216.02. The Views of the manufacturers are not necessarily the official views of, or endorsed by, the U.S. Government, the Department of Defense, or the Department of the Air Force. No Federal endorsement of manufacturers is intended.

## Figures and Tables

**Table 1:** Participant demographic characteristics.

	Overall (*n* = 72)	Experiment 1 (*n* = 42)	Experiment 2 (*n* = 30)
**Age, years, Median [IQR]**	33.0 [28.0, 38.0]	31.0 [28.2, 36.8]	35.5 [27.2, 42.0]
**Sex, *n* (%)**			
Male	37 (51.4)	22 (52.4)	15 (50.0)
Female	35 (48.6)	20 (47.6)	15 (50.0)
**Military Status, *n* (%)**			
Active Duty	54 (75.0)	40 (95.2)	14 (46.7)
Guard	11 (15.3)	1 (2.4)	10 (33.3)
Reserve	7 (9.7)	1 (2.4)	6 (20.0)
**Job Role, *n* (%)**			
Nurse	34 (47.2)	18 (42.9)	16 (53.3)
Physician	20 (27.8)	14 (33.3)	6 (20.0)
Respiratory Therapist	17 (23.6)	10 (23.8)	7 (23.3)
Paramedic	1 (1.4)	0 (0.0)	1 (3.3)
**Years of Practice, Median [IQR]**	4.0 [3.0, 7.0]	4.0 [3.0, 5.0]	4.5 [3.0, 13.8]
**Prior CCAT Training, *n* (%)**			
Yes	31 (43.1)	12 (28.6)	19 (63.3)
No	41 (56.9)	30 (71.4)	11 (36.7)

**Table 2:** Data quality and retention rates.

	HRV Data	Motion Data
**Data Collection**		
Windows (2-min) collected	2301	2207
Subjects	72	72
Subject-periods *	162	162
**After Quality Control**		
Windows retained	2131 (92.6%)	2197 (99.5%)
Subjects retained	69 (95.8%)	72 (100%)
Subject-periods retained	148 (91.4%)	162 (100%)
**Quality Control Exclusions**		
Level 1: Windows processing failures	57 (2.5%)	10 (0.5%)
Level 2: Poor quality windows	88 (3.9%) **	N/A
Level 3: Subject-period exclusions	6 subject-periods ***	N/A

* Subject-periods = unique combinations of Subject × Period (Baseline, Scenario 1A, Scenario 2A, Scenario 2B); ** Percentage of Level 1 successful windows; *** Excluded subject-periods: 3 Baseline, 2 Scenario 1A, 1 Scenario 2B.

**Table 3:** HRV metrics (baseline vs. simulation).

	Baseline Median [IQR]	Simulation Median [IQR]	% Change	Unadjusted Model	Adjusted Model *
**RMSSD (ms)**	34.4 [19.9, 56.3]	20.7 [12.7, 29.1]	−39.7%	β = −16.32, *p* < 0.001	β = −16.98, *p* < 0.001
				**d = −0.58**	**d = −0.59**
**LF/HF Ratio**	3.78 [2.27, 6.10]	5.83 [4.41, 8.05]	+54.2%	β = 1.85, *p* < 0.001	β = 1.81, *p* < 0.001
				**d = 0.56**	**d = 0.54**
**Heart Rate (bpm)**	80.9 [70.4, 92.6]	104.3 [91.6, 116.1]	+29.0%	β = 24.32, *p* < 0.001	β = 24.89, *p* < 0.001
				**d = 1.22**	**d = 1.22**

* Adjusted for Age, Sex, prior CCAT Training, and Experiment.

**Table 4:** Motion metrics (baseline vs. simulation).

	Baseline Median [IQR]	Simulation Median [IQR]	Test Statistic	*p*-Value	Effect Size (r)
**Acc SD (m/s^2^)**	0.000 [0.000, 0.015]	0.143 [0.114, 0.170]	W = 11	*p* < 0.001	**r = 0.94**
**Acc Max (m/s^2^)**	0.000 [0.000, 0.163]	0.913 [0.859, 0.971]	W = 13	*p* < 0.001	**r = 0.94**
**Speed Mean (km/h)**	0.000 [0.000, 0.010]	0.163 [0.101, 0.229]	W = 1	*p* < 0.001	**r = 0.97**
**Speed SD (km/h)**	0.000 [0.000, 0.043]	0.581 [0.428, 0.736]	W = 7	*p* < 0.001	**r = 0.92**
**Speed Max (km/h)**	0.000 [0.000, 0.700]	3.430 [3.088, 3.770]	W = 33	*p* < 0.001	**r = 0.92**
**Distance (m)**	0.000 [0.000, 0.200]	4.950 [2.895, 6.928]	W = 21	*p* < 0.001	**r = 0.94**
**Cadence Active (%)**	0.000 [0.000, 0.000]	6.667 [4.167, 8.750]	W = 0	*p* < 0.001	**r = 1.00**

**Table 5:** Baseline-adjusted RMSSD correlations with NASA-TLX.

NASA-TLX Dimension	β Simulation	95% CI Simulation	*p*-Value Simulation	β Baseline	95% CI Baseline	*p*-Value Baseline
**Overall Workload**	**−0.269**	[−0.471, −0.067]	**0.009**	**+0.523**	[0.165, 0.881]	**0.004**
**Mental Demand**	**−0.25** **0**	[−0.424, −0.075]	**0.005**	**+0.483**	[0.171, 0.795]	**0.002**
**Temporal Demand**	**−0.233**	[−0.385, −0.080]	**0.003**	**+0.434**	[0.160, 0.709]	**0.002**
Physical Demand	−0.184	[−0.387, 0.020]	0.077	+0.080	[−0.279, 0.439]	0.661
Effort	−0.103	[−0.298, 0.092]	0.300	+0.225	[−0.118, 0.568]	0.197
Frustration	−0.157	[−0.375, 0.062]	0.160	+0.383	[−0.003, 0.769]	0.052
Performance	−0.088	[−0.299, 0.122]	0.411	+0.325	[−0.047, 0.697]	0.087

Model: NASA_TLX ~ Simulation_RMSSD + Baseline_RMSSD + (1|Subject). β_simulation = standardized effect of simulation RMSSD after controlling for baseline (tests reactivity). β_baseline = standardized effect of baseline RMSSD (individual trait differences).

**Table 6:** Relationships between HRV and Motion metrics during simulation.

HRV Metric	Motion Metric	Spearman ρ	LMM β	95% CI	*p*-Value
**RMSSD**	Acc SD	−0.089	−0.052	[−0.184, 0.080]	0.441
	Acc Max	+0.021	+0.048	[−0.083, 0.179]	0.472
	Speed Mean	−0.156	−0.103	[−0.251, 0.045]	0.171
	Speed SD	−0.178	−0.127	[−0.284, 0.030]	0.111
	**Speed Max**	**−0.243**	**−0.178**	**[−0.326, −0.030]**	**0.019** *
	Distance	−0.201	−0.145	[−0.303, 0.013]	0.072
	Cadence Active	−0.167	−0.119	[−0.275, 0.037]	0.134
**Heart Rate**	Acc SD	+0.134	+0.096	[−0.036, 0.228]	0.152
	Acc Max	+0.089	+0.067	[−0.064, 0.198]	0.315
	**Speed Mean**	**+** **0.267**	**+** **0.221**	**[0.086, 0.356]**	**0.001** *
	**Speed SD**	**+** **0.245**	**+** **0.203**	**[0.065, 0.341]**	**0.004** *
	**Speed Max**	**+** **0.289**	**+** **0.246**	**[0.113, 0.379]**	**<0.001** *
	**Distance**	**+** **0.312**	**+** **0.268**	**[0.137, 0.399]**	**<0.001** *
	**Cadence Active**	**+** **0.228**	**+** **0.191**	**[0.053, 0.329]**	**0.007** *
**LF/HF Ratio**	Acc SD	+0.067	+0.041	[−0.091, 0.173]	0.543
	Acc Max	+0.045	+0.028	[−0.103, 0.159]	0.674
	Speed Mean	+0.112	+0.079	[−0.069, 0.227]	0.293
	Speed SD	+0.089	+0.063	[−0.089, 0.215]	0.420
	Speed Max	+0.134	+0.098	[−0.034, 0.230]	0.145
	Distance	+0.156	+0.118	[−0.020, 0.256]	0.093
	Cadence Active	+0.098	+0.071	[−0.081, 0.223]	0.358

* *p* < 0.05.

**Table 7:** Experiment 1 vs. Experiment 2 comparison: significant findings.

Biomarker Domain	Metric	Experiment 1	Experiment 2	Difference	95% CI	*p*-Value	Cohen’s d
**HRV Reactivity**							
	RMSSD Change	−36.1% (31.9 → 20.4 ms)	−54.4% (46.8 → 21.3 ms)	β = −14.38	[−24.43, −4.32]	**0.005** *	**−0.5** **0**
	Heart Rate Change	+13.3% (87.0 → 98.6 bpm)	+40.4% (75.9 → 106.6 bpm)	β = 9.30	[3.65, 14.94]	**0.001** *	**0.45**
	LF/HF Ratio Change	+47.8%	+76.7%	β = 0.25	[−1.12, 1.62]	0.72	0.08
**NASA-TLX**							
	Overall Workload	32.6 [26.7, 42.7]	45.0 [34.8, 57.7]	β = 8.41	[1.19, 15.63]	**0.022** *	**0.5** **0**
	Mental Demand	41.2 [26.5, 52.9]	60.0 [33.8, 78.1]	β = 10.98	[0.37, 21.60]	**0.043** *	**0.46**
	Temporal Demand	38.2 [23.5, 52.9]	60.0 [28.8, 80.0]	β = 16.06	[3.86, 28.25]	**0.010** *	**0.56**
	Performance †	29.4 [17.7, 39.7]	40.0 [20.0, 60.0]	β = 11.86	[2.40, 21.33]	**0.014** *	**0.54**
	Physical Demand	17.7 [8.8, 23.5]	20.0 [5.0, 40.0]	β = 2.91	[−5.93, 11.74]	0.519	0.14
	Effort	42.7 [30.9, 52.9]	50.0 [30.0, 71.3]	β = 3.87	[−6.53, 14.27]	0.466	0.17
	Frustration	22.1 [11.8, 37.5]	20.0 [2.5, 60.0]	β = 5.53	[−5.60, 16.67]	0.330	0.22
**Motion**							
	Acc SD (m/s^2^)	0.144 [0.116, 0.174]	0.147 [0.123, 0.170]	β = −0.006	[−0.024, 0.012]	0.506	−0.16
	Acc Max (m/s^2^)	0.940 [0.861, 1.061]	0.900 [0.857, 0.926]	β = −0.061	[−0.112, −0.010]	**0.018** *	**−0.58**
	Speed Mean (km/h)	0.166 [0.080, 0.260]	0.166 [0.122, 0.209]	β = −0.005	[−0.048, 0.038]	0.812	−0.06
	Speed SD (km/h)	0.643 [0.411, 0.797]	0.554 [0.455, 0.642]	β = −0.095	[−0.193, 0.004]	0.061	−0.46
	Speed Max (km/h)	3.553 [3.233, 3.987]	3.282 [3.025, 3.463]	β = −0.390	[−0.668, −0.113]	**0.006** *	**−0.65**
	Distance (m)	5.486 [2.675, 8.504]	4.600 [3.025, 6.025]	β = −0.929	[−2.169, 0.310]	0.142	−0.35
	Cadence Active (%)	6.875 [3.854, 9.167]	6.250 [4.896, 8.333]	β = −0.459	[−1.830, 0.912]	0.512	−0.16

Values shown as Median [IQR] unless otherwise noted. † Performance reverse-scored: higher values = worse perceived performance. * *p* < 0.05.

**Table 8:** Summary of multimodal task workload assessment.

Metric	Captures	Independent of Movement?	Convergent Validity with NASA-TLX	Convergent Validity with HRV
**RMSSD**	Parasympathetic activity	**Yes** (1 of 7 motion correlations significant)	**Yes** (after baseline adjustment)	N/A
**Heart Rate**	Cardiovascular activation	**No** (5 of 7 motion correlations significant)	**No** (unadjusted or baseline-adjusted)	N/A
**LF/HF Ratio**	Sympathovagal balance	**Yes** (0 of 7 motion correlations significant)	**No** (unadjusted or baseline-adjusted)	N/A
**Motion**	Physical activity patterns	**No**	**No** (0 of 49 motion correlations significant)	**Yes** (moderate with HR)

“Independent of Movement?” reflects correlations between each metric and motion during simulation. “Convergent Validity with NASA-TLX” indicates significant associations with subjective workload dimensions. “Convergent Validity with HRV” shows associations between motion and HRV indices (RMSSD, Heart Rate, LF/HF Ratio).

## Data Availability

The raw data collected in this study are not publicly available due to privacy and ethical policies and regulations.

## Supplementary Materials

The following supporting information can be downloaded at: https://www.mdpi.com/article/10.3390/s26113596/s1.
