# Supplementary material for: Heart Rate Variability and Body Motion as Digital Biomarkers of Task Workload During Military En Route Critical Care Simulations
Source: Sensors (Basel). 2026 Jun 5;26(11):3596. doi: 10.3390/s26113596 (PMC13259423; doi:10.3390/s26113596)
Supplement: Supplementary file 1 [file sensors-26-03596-s001.zip › Supplementary materials file S1.pdf]

Supplementary materials file S1

Phase 1 Patient Simulated Scenarios for Participants

Name Chuck (TBI) **Age** 26 **M** **Allergies** NKDA **Wt** 80 kg **Ht** 70 in

MOI/Injuries:
Mounted IED Blast 1 day ago.
Severe TBI, initial GCS 3.

Tubes/Lines/Drains: R radial arterial line; R subclavian TLC; codman bolt ICP monitor; OG tube; foley

VS: T _102__ HR _110__ BP _115_ /_70_ (85); ICP: 15; CPP: 70 RR _16_ SpO2 _99_%

I/O: (24Hr) __1.2 L_ /_0.5 L_ ***Blood***: None received

CBC: HGB _10_ HCT 30__ WBC _4__ Plt 150

BMP: Na_140 Cl 105_ BUN _8_ K___4___CO2_22_ Cr_0.8_

Renal: Ca 1.2

Coags: INR _1.0_

NEURO: Initial GCS: 3; current neuro: 6T off sedation; withdraws RUE/RLE; Pupils- PERRL
Head CT: Intraparenchymal hemorrhage; no shift; ICP monitor (bolt) in place
Spine: C-collar in place; cleared for 30 degrees head of bed
ICP Monitoring: Initial ICP: 15; current ICP 15; ICP has been stable; Codman Reference#: 999
Meds: Seizure prophylaxis – Keppra 500mg; no Tylenol or 3% NS given
Neurosurgery recommendations: Follow TBI CPG for all goals; not cleared for anticoagulation; no altitude restriction; no other recommendations

PULM: ***ABG***: (6 hours ago) pH 7.39__ pCO2 _43__ pO2 109__ HCO3 __24_ BD __-2_ SpO2 99%__
Vent: AC / VC Vt _440__ RR __18__ PEEP _5__ FiO2 _40%_

ETT: 7.5, 22 cm at teeth | CXR: stable (2 hours ago)

CVS: Sinus rhythm without ectopy

ABD/GI/GU: CT Negative; No injuries identified

MSK: 2+ pulses in all 4 extremities; no significant injuries identified

PPI: GI—Protonix given 2 hours ago | **VTE:** held per NSRG recs **ID:** Antibiotics—Ancef given one hour ago

DRIPS: ***Sedation***: Propofol 50 mcg/kg/min; ***Maintenance fluid:*** NS 100 cc/hr

Name Davie (Ex-fix) **Age** 24 **F** **Allergies** NKDA **Wt** 70 kg **Ht** 70 in

Injuries/Course:
Mounted IED blast 1 day ago with open Right leg tib/fib fracture from vehicle rollover. No other injuries identified.
Ex-fix placed on right leg. Procedure completed approximately 30 minutes ago. CCATT required due to ongoing pain.

Tubes/Lines/Drains: R arm 16 g PIV x1; L radial arterial line

VS: T _99_ HR _98__ BP _106_ / _68 (81) RR _20_ O2 _98_%

I/O: (24Hr) __2.5__ / _0.8__ ***Blood***: RBCs __2 (given in the operating room, no recent requirements)

CBC: HGB _9__ HCT 26__ ***BMP:*** Na 140_ Cl 110 BUN 12__ **Other:** iCa 1.2 INR 0.8 Lactate**:** 2.3
WBC _9__ Plt _150_ K _3.8_ CO2_18_ Cr_1.2_

NEURO: Initial GCS:15; current GCS: 15; no deficits
Head CT: Negative
Spine: Cleared (cervical, thoracic and lumbar spine all cleared)
Pain: Requiring IV pain control; last given 1.0 mg dilaudid just prior to transfer to aircraft

PULM: 98% on room air at sending facility
ABG: (1 hour ago): pH _7.35 pCO2 _42__ pO2 _128_ HCO3 _18_ BD __-3_ SpO2 _98%
CXR: (6 hours ago): clear bilaterally

CVS: Sinus rhythm without ectopy

ABD/GI/GU: CT Chest/abdomen/pelvis: Negative; no injuries identified
NPO—Patient recently out of surgery; still recovering from anesthesia

MSK: Ex-fix on right leg; 2+ pulses distally; good distal perfusion; requiring CCATT for pain control requirements

Prophylaxis: GI: **Protonix** 1 hour ago VTE: **Lovenox** 1 hour ago

ID: Antibiotics- **Ancef** 4 hours ago

DRIPS: No current drips

Supplemental Material: Phase 1 Patient Summaries for Instructors

Simulation Patient #1 – Chuck TBI

Clinical Summary (TBI)

**DX/PHASE OF CARE**	Trauma / Time since POI: 16 hours / Post-surgery, Ongoing Critical Care		
**PATIENT MOVEMENT**	Role 2 (w/CCATT) to Role 3	**EVACUATION TYPE**	TACEVAC

HISTORY OF PRESENT ILLNESS:

26yo Male, 80kg, post injury day #1; mild improved explosive device Blast Injury w/ closed TBI (initial GCS 3T); CT chest/Abd/Pelvis negative/ Intraparenchymal Hemorrhage (IPH) noted on CT head, no shift, w/ bolt, intubated. Decompressing hospital w/ CCATT. Critical Care monitoring initiated.

LAST 8 HOURS:

- GCS now 6T (withdraw RUE and RLE). Requiring increased sedation for goal Richmond Sedation Score (RASS) -4 (Deep Sedation).
- Intercranial Pressure (ICP) 15 while sedated.
- SpO2 99%, $ETCO_2$=41.
- Current ABG: 7.39/43/109/24/-2.
- No ventilator changes in last 6 hrs. ACVC TV 500 (~6mL/kg), RR 18, PEEP 5, FiO2 40%.
- Urine output stable: 50cc/hr for last 4 hrs.
- Temp 102F.
- Keppra and Ancef prophylaxis given.
- Neurosurgery states follow CPGs, do not give Lovenox.

PROBLEM LIST:

- TBI (Severe) – IPH w/o shift, no skull fracture
- Hyperthermic

CARE PLAN:

TBI – PRIMARY OBJECTIVES

- Maintain Cerebral Perfusion (CPP 60-80)
- Recognize intracranial hypertension and follow treatment algorithm
- Appropriately prioritize primary objectives over secondary objectives

SEDATION:

- Current: Propofol @ 50 mcg/kg/hr and fentanyl @ 100 mcg/hr
- Neuro Checks q1h
  - Goal: RASS -4
- Discontinue propofol for hypotension
  - Consider: Fentanyl 0.5mcg/kg slow IVP or 25-250 mcg/hr; Ketamine 0.1-0.3 mg/kg IVP or 0.1-0.3 mg/kg/hr; Versed

Other primary treatment objectives are met without intervention

1) Avoid hypoxia- Goal: SpO2 > 93% | PaO2 > 80
2) Coagulopathy Not Present (Goal INR ≤ 1.3)

TBI – SECONDARY OBJECTIVES

- Manage ventilation to meet pCO_2 goals
- Treat hyperthermia
- Consider 3% NS for sodium goals

VENTILATION

- Goal pCO_2 35-40

NORMOTHERMIA:

- Goal: Temp < 98.6°F; Address increased temperature
  - Tylenol 1 GM or 'cool aircraft'

THERAPEUTIC HYPERNATREMIA:

3 % Saline (Hypertonic) Protocol

- Goal: Na+ level (150-160)
- 3% Saline bolus 250 ml, then 50-100 mL/hr

PROPHYLAXIS (PPX): All already given

- Keppra 500mg every 12hrs
- GI: Protonix 40mg IV/PO Daily
- VTE: not cleared for lovenox per neurosurgery; SCDs unavailable to CCATT
- Antibiotics for ICP monitor: cefazolin 2 GM Q 6-8hrs

LINES/TUBES/DRAINS: SC TLC, Radial A-line, L-AC PIV, Bolt, ETT, OGT, Foley

SIMULATION TIMELINE: 10 minutes

- Handoff is received from the sending team prior to taking over patient care aboard the aircraft.

00:00 – 02:00 minutes:

- Team enters the scenario when plane is at altitude and team is free to move around the cabin. Team begins post-takeoff assessments.
- At 90 seconds, patient begins transition from baseline state to intermediate state demonstrating a worsening sedation level and increased intercranial pressure (ICP). These are indicated by increased HR, BP, RR, ICP on the monitor.

02:00 – 04:00 minutes: Increased ICP event

- Patient has increased ICP due to inadequate sedation.
- Participants are expected to recognize that increased ICP is present, appropriately prioritize this as an emergency, and follow the increased ICP algorithm. The first step is to assess pain control and sedation. Team is expected to recognize that inadequate sedation is the cause of the increased ICP event.
- The critical decision making in selecting a sedative is to recognize that CPP is 60. Given the primary objective of CPP 60-70, propofol is inappropriate. The team must select and administer a sedative.

04:00-07:00

- If no sedative given by 4 minutes, the confederate states over the headset, "Your TBI patient is waking up." This is a verbal queue to ensure forward progression of sim for teams which are slow to recognize the event. If no acknowledgement by 04:30, confederate states "Your TBI patient is waking up, please confirm you hear me."
- If propofol given, ICP improves but BP drops so patient transitions to low CPP state.
- If blood pressure sparing sedative given (ex: Ketamine/Fentanyl/Versed), then ICP event resolves and CPP remains at goal.

07:00-10:00
Branchpoint 1: ICP event treated; CPP at goal

- Team is expected to assess and treat secondary objectives of normothermia and therapeutic hypernatremia.

Branchpoint 2: Low CPP state

- If propofol is given, patient's CPP decreased below goal. Team must recognize low CPP, appropriately prioritize this as an emergency, and follow the cerebral hypoperfusion algorithm. Best actions include vasopressor administration (IV push or drip); reducing propofol dose and providing alternative sedative. Alternative actions include fluid boluses (3% NS, NS, blood) given that patient is well-resuscitated based on available data.
- If low CPP is resolved before 10:00, then team is expected to move onto secondary objectives.

TBI MANAGEMENT LEARNING OBJECTIVES:

- ✓ Assess and maintain cerebral perfusion pressure (CPP) as top priority.
- ✓ Recognize increased intracranial pressure and follow treatment algorithm.
- ✓ Appropriately prioritize cerebral perfusion over secondary treatment objectives of temperature, carbon dioxide and sodium goals.

RELEVANT CPGS:

- Neurosurgery and Severe Head Injury
- Mechanical Ventilation
- Pain, Anxiety, Delirium

Simulation Patient #2 'Davie'- Extremity Fracture

Clinical Summary (Extremity Fracture)

**DX/PHASE OF CARE**	Trauma / Time since POI: 16 hours / Post-surgery, Ongoing Critical Care		
**PATIENT MOVEMENT**	Role 2 (w/CCATT) to Role 3	**EVACUATION TYPE**	TACEVAC

HISTORY OF PRESENT ILLNESS:

24yo Male, 90kg; s/p Mild IED Blast Injury w/ RLE Tib/Fib Post External Fixator (Ex-Fix). Pelvis negative by scan. Transfused 2 PRBC. Patient has been given Ancef, Lovenox, Proton pump inhibitors (PPI).

HOSPITAL COURSE: Pt had presented with open Tib/Fib. Went to OR for wash out and EX-Fix. Then in ICU for pain management. Now packaged and loaded to aircraft by other CCAT team. Pending conversion to ORIF at Role 3.

LAST 12 HOURS:

- As above.
- Pt reports continuous 4/10 pain and leg are sensitive to touch, but tolerable.
- Pulses are present and strong in BLE.
- Temp: 98.
- No redness at surgical sites.
- Current ABG: 7.35/42/128/18/-3. Lactate 1.5. Hgb 9. Plt 200k.

PROBLEM LIST:

- Pain and pain control
- Somnolence after analgesia
- Hypoxia

CARE PLAN:

Primary Objectives:

- Recognize pain and appropriately administer analgesic
- Recognize hypoxia and provide supplemental oxygen

ANALGESIA

- Patient is NPO after surgery. Appropriate medication options include Fentanyl; Morphine; Ketamine; Hydromorphone
- Team expected to appropriately follow universal precautions using alcohol wipes as needed

RESPIRATORY

- Patient will experience hypoxic event. Team expected to provide supplemental oxygen with appropriate connections to aircraft oxygen.

Secondary Objectives:

- Crew Resource Management

GENERAL

- Team must balance crew resources and maintain awareness of this less critical patient while meeting objectives for the critically ill patient with TBI

SIMULATION TIMELINE: 10 minutes

- Handoff is received from the sending team prior to taking over patient care aboard the aircraft.

00:00 – 02:00:

- Best practice is for CCAT team to send one participant to evaluate Dave.
- Live role player (LRP) has worsening pain due to vibration during take-off. LRP requests pain medication and rates pain as 10/10 if asked.

02:00 – 06:00: State of increased pain

- The team are expected to perform as a team and complete tasks for assessing a patient with extremity fracture who is experiencing uncontrolled pain due vibration during take-off, and then administer appropriate analgesic medication.
- HR and BP increase on LRP's monitor if no pain meds given. LRP repeats request for pain meds if asked.
- An IV line will be present and strapped onto the LRP patient's arm enabling participants to perform the process of IV medication administration.
- The team must select an appropriate analgesic medication and administer the medication. Note that team resource management is needed to meet this objective while simultaneously addressing the higher priority of increased ICP for Chuck.

06:30– 10:00: Hypoxia event

- Patient transitions to hypoxic state at 6:30 whether or not pain medication is provided.
- LRP patient closes eyes and oxygen saturation decreases to 87%. Will open eyes if asked.
- CCATT is expected to provide supplemental oxygen by placing patient on either nasal cannula or face mask and appropriately connecting to aircraft oxygen.
- CCATT is expected to reassess patient's SaO_2 level.

PAIN MANAGEMENT LEARNING OBJECTIVES:

- ✓ Recognize increased pain and appropriately administer an appropriate analgesic medication
- ✓ Recognize changes in clinical status and treat hypoxia with supplemental oxygen

RELEVANT CPGS:

- Pain, Anxiety, Hypoxia

Supplemental Material: Phase 2, Sim A Patient Summaries for Participants

Julie Caldwell // DOB: 3 Apr 2004 // NKDA **Current Day: JD100**

Patient history:
Pt is a 20 y/o female, s/p dismounted vehicle-born IED on JD098. Pt arrived intubated. Injuries include: 1) R parieto-occipital skull fx and 2) left tempoparietal intraparenchymal hemorrhage. CT of the head revealed a 1.5cm right posterior temporal contusion and a right orbitofrontal contre-coup contusion. Pt went to OR on JD99 for placement of Codman ICP monitor. EBL 30mL. Pt transferred to the ICU postop and is stable. Last GCS 6 and remains intubated. Repeat CT showed no new or enlarging intracranial hemorrhage. No new mass effect or midline shift. Patient receiving 3% sodium solution IV per protocol to maintain Na of 150-160 for ICP control. ICP has been 10-16. Pt also has C-collar for transport.

Neurosurgery recs: Will not need altitude restrictions (small amount of intraparenchymal air). Follow TBI CPG. Not cleared for DVT ppx.

Drug allergies: NKDA | **Height** 67 inches | **Weight** 55 kg

Temp 102.0°F; **BP** 140/70; **MAP** 84; **HR** 104; **RR** 18; **SpO2** 99%, **ETCO2** 47; **ICP** 16; **CPP** 68

Labs: (2 hours ago): WBC 10; Hgb 12.1; Hct 36.5; PLT 153K; Na 148; PT 14.9; INR 1.1
pH 7.37; **pCO2** 47; **pO2** 115; **HCO3** 20; **BE** -5; Lactate 2.4

Rads: CT head: Comminuted right skull fracture extending into the right temporal bone. No new or enlarging intracranial hemorrhage.
CT of chest/abd/pelvis: negative for injury.
CXR (1 day ago): ETT in good position.

Ventilation mode: VC, **FiO2** 40, **TV** 370, **Rate** 18, **PEEP** 5
No changes in the past 12 hours. Minimal secretions. 7.5 ETT, 23 at teeth.

Access: Triple Lumen Catheter (TLC) (Right Subclavian); A-Line (Right Arm)

Drips: Propofol 30 mcg/kg/min, Fentanyl 100mcg/hr, 3% NS 50ml/hr

Scheduled Medications: Keppra 500mg q12h (**last dose 13h ago**); cefazolin 2gm Q 8h (**last dose 9h ago**)

Drainage: Indwelling Catheter (Bladder) Gravity; Gastric Tube (Oral) Intermittent;

PMI Data: Backrest; Litter Pad; Cardiac Monitor; Pulse Ox; IV Pump; Suction; Ventilator; Wound Vac; Codman Monitor

Other Equipment: Codman ICP monitor, Keep HOB at 30deg (load head 1st); C-Collar

Diet: NPO-Nothing By Mouth

Brian Grover // Allergy: Codeine // DOB: 8 Jul 2000 **Current Day: JD100**

Patient History:
24 y/o Male, s/p IED Blast w/Blunt Trauma to Abd with a positive FAST exam on JD098. CT of head and spine cleared; Pt arrived intubated. Went to OR on JD099 for Ex-Lap. Post-op CT showed extensive liver lac, s/p surgery w/extensive surgical packing. Temporary abdominal closure (TAC) placed to suction 125mmMg. 4 u pRBC, 4 u FFP, 1 u platelets, 1 U whole blood given during case. MAP currently 65. Last given 1 U pRBC 6 hours ago. Not cleared for DVT ppx.

Drug Allergies: Codeine

Temp 99.7°F; **BP** 90/55 **MAP** 63; **HR** 125; **RR** 16; **SpO2** 95%; **ETCO2** 42

Height: 72 inches **Weight:** 92kg

Labs (2 hours ago): Hgb 7, Hct 21, WBC 12.2, PLT 103
pH 7.31, pCO2 42, pO2 99, HCO3 18, BE -7, Lactate 4.2
NA 146, K 4.2, CL 115, CO2 18, BUN 18, CR 1.6, GLUCOSE 118
PT/INR/PTT 14.4/1.4/40.4

Rads: CT head/c-spine: negative
CXR: ETT in good position.

Ventilation Mode: AC **FiO2** 40% **TV** 466 **RR** 18 **PEEP** 5 **PIP** 10
No changes in the past 12 hours. Minimal secretions. 7.5 ETT, 23 at teeth.

Access: Triple Lumen Catheter (TLC) (Right Subclavian); A-Line (Right Arm); Saline Lock (Left Arm)

Drips: Ketamine 46mg/hr (0.5mg/kg/hr); Fentanyl 150 mcg/hr; Whole blood
Scheduled Medications: ancef 1 gm IV q6h (last dose **8 hours ago**), flagyl 500 mg IV q6h (last dose **8 hours ago**), Protonix 40 mg IV Q24h (last dose 8 hours ago).

Drainage: Indwelling Catheter (Bladder) Gravity; Gastric Tube (Oral) Intermittent; TAC to suction -125mm

Ortho:

PMI Data: Backrest; Litter Pad; Cardiac Monitor; Pulse Ox; IV Pump; Suction; Ventilator; Wound Vac

Other Equipment:

Feeding Tube: NGT

Diet: NPO-Nothing By Mouth

Molly Bailey // DOB: 9 May 2002 // NKDA **Current Day: JD100**

Patient History:
Pt is a 22 y/o female who was in a vehicle fire and sustained an inhalation injury. TBSA 40% burns. Patient presented with burns to the body including 3rd degree to the RLE and face, and 2nd degree to the buttock and low back. Intubated for airway protection. Patient was taken to the CT scanner followed by OR for debridement, and RLE escharotomy. In the OR pt was stable with pressors and making good urine. Patient also underwent bronchoscopy and found to have inhalation injury. No TBI or abdominal injury. CT head/chest/abdomen/pelvis negative for injury. Time since injury is 12 hours. See attached burn flow sheet for resuscitation. LR resuscitation ongoing at 850 ml/hr. Total IVF 7 L. Levophed started 4 hours ago.

Drug allergies: NKDA | **Height** 68 inches | **Weight** 65Kg

Temp 99.0F; **BP** 70/45; **MAP** 53; **HR** 132; **RR** 23; **SpO2** 94%; **ETCO2** 41

Labs: (2 hours ago**)** WBC 16; Hgb 14; Hct 40; PLT 153K;
NA 129, K 4.2, CL 103, CO2 19, BUN 31, CR 2.3, GLUCOSE 118;
pH 7.3; **pCO2** 39.5; **pO2** 73; **HCO3** 19; **BE** -6, Lactate 4.8

Rads: CXR (1 day ago): ETT in good position. Bilateral patchy airspace disease
CT of head/c-spine/chest/abd/pelvis: negative for injury.

Ventilation mode: AC, **FiO2** 50%, **TV** 410, **Rate** 18, **PEEP** 8
No changes is the past 12 hours. Moderate secretions. 8.0 ETT, 23 at teeth. Received heparin and albuterol neb 6 hours ago.

Access: Triple Lumen Catheter (TLC) (Left Subclavian); A-Line (Left Arm)

Drips: Propofol 50mcg/kg/min; Fentanyl 200mcg/hr; Levophed 5 mcg/min; LR 850 ml/hr

Scheduled Medications: Meropenem 1 gm IV q8h (last dose **10 hours ago**); Lovenox 30 mg SC q 12 hours (last dose **15 hours ago**); Protonix 40 mg IV q24h (last dose 12 hours ago)

Drainage: Nasogastric Tube (R-Nare) Intermittent; Indwelling Cath (Bladder) Gravity

PMI Data: Backrest; Litter Pad; Cardiac Monitor; Pulse Ox; IV Pump, Suction; Ventilator

Other Equipment: None

Diet: NPO-Nothing By Mouth

Admin Remarks: None

Stan Kim //   DOB: 13 Aug 2003   // NKDA   **Current Day: JD100**

Patient History:
21yo M, 70kg, s/p dismounted IED blast w/ sustained polytrauma JD096. Right lower leg open fracture, degloving of entire right lower limb, pelvic fractures, injury to right iliac vessels, urethral injury with bladder displacement. Taken to OR on JD096 debridement of all wounds, pelvic x-fix, right leg with dressings in place, placement of supra-pubic catheter and vessel repair. Patient received blood products: 40 PRBC, 40 FFB, 10 whole blood, 5 platelet. Patient taken to ICU intubated. Has been on ancef. Has had stable postoperative course with stable hemoglobin x24 hours and no further blood product requirement. Developed fever 12 hours ago.

Drug allergies: NKDA | **Height** 69 inches | **Weight** 70 kg

Temp 101.0°F; **BP** 113/64 **MAP** 80; **HR** 90; **RR** 16; **SpO2** 99%

Labs: (2 hours ago): WBC 17; Hgb 11.8; Hct 35; PLT 201K;
NA 140, K 3.9, CL 111, CO2 22, BUN 19, CR 1.1, GLUCOSE 158;
pH 7.35; **pCO2** 36; **pO2** 118; **HCO3** 18; **BE** -5; Lactate 2.6

Rads: CXR (1 day ago): ETT in good position.
CT head/c-spine/chest: negative for injury.
CT of chest/abd/pelvis: Pelvic fracture, bladder injury, right iliac artery and vein injury.

Ventilation mode: AC, **FiO2** 30%, **TV** 424 **Rate** 16, **PEEP** 5
No changes in the past 12 hours. Minimal secretions.

Access: Triple Lumen Catheter (TLC) (Right Subclavian); A-Line (Right Arm)

Drips: Ketamine 34mg/hr (0.5mg/kg/hr); LR 150ml/hr

Scheduled Medications: Ancef 1 gm IV q8hrs (last dose **10 hours ago**); Protonix 40 mg IV Q24h (last dose 6 hours ago); Lovenox 40 mg SC q12hrs (last dose **13 hours ago**).

Drainage: Nasogastric Tube (R-Nare) Intermittent; Suprapubic (lower abdominal area) Gravity

Ortho: Ex-Fx (Pelvis), C-collar

PMI Data: Backrest; Litter Pad; Cardiac Monitor; IV Pump; Suction; Ventilator

Other Equipment: Suprapubic Cath; A-Line

Feeding Tube: NGT

Diet: NPO-Nothing by Mouth

George Smith // DOB: 1 Sep 1994 // NKDA **Current Day: JD100**

This is your live role player. The patient may be male or female depending on staff availability.

Patient History:
Pt is 30 yo. Presented to Role 3 on JD 96 s/p VBIED. GCS on admit was 15. Injuries included 1) comminuted R tib/fib fx-- s/p ExFix. Pt went to OR JD96 for ex/fix right leg. Pt was extubated and is stable off the vent. Current H/H is :9.8/27.7 .

UPDATE JD 99: pt was diagnosed with a PE. Pt was started on IV heparin. Transfer for IVC filter. EKG with no ischemic changes.

Drug allergies: NKDA | **Height** 74 inches | **Weight** 86 kg

Temp 98.0°F; **BP** 152/73; **HR** 104; **RR** 18; **SpO2** 96% on room air

Labs: (2 hours ago): WBC 8; Hgb 9.8; Hct 27.9; PLT 201K
NA 142, K 4.1, CL 112, CO2 25, BUN 18, CR 1.0, GLUCOSE 125
pH 7.33; **pCO2** 42; **pO2** 75; **HCO3** 25.6; **BE** 0
PT 14.9; INR 1.1; aPTT 80

Rads: CT head/c-spine: negative. Initial CT chest/abd/pelvis: negative. Repeat CTA chest: pulmonary embolism involving right pulmonary artery.

Ventilation mode: N/A

Access: Triple Lumen Catheter (TLC) (Left Subclavian); A-Line (Left Arm)

Drips: heparin 1500 units/hr ggt

Scheduled Medications: Dilaudid 1 mg IV q6 hours (last dose **8 hours ago**)

Drainage: n/a

Ortho: External Fixator (Right Short Leg)

PMI Data: Backrest; Litter Pad; Cardiac Monitor; Pulse Ox; IV Pump; Suction; Ventilator

Other Equipment:

Feeding Tube: N/A

Diet: Regular

Supplemental Material: Phase 2, Sim A Patient Summaries for Instructors

Julie Caldwell
Clinical Summary (TBI)

Scenario States, Modifiers and Triggers				
Patient State/Vitals	Patient Status	Learner Actions, Modifiers and Triggers to Move to Next State		Facilitator Notes
**1. Baseline State**<br>Rhythm: Sinus<br>HR: 104<br>BP: 125/65<br>RR: 18<br>O2SAT: 99%<br>T: 102° F<br>GCS: 6T<br>MAP: 85<br>CPP: 69<br>ETCO2: 47	Pt intubated and sedated, GCS 6T (withdrawing UP and LE). ICP monitor in place, head wrapped.	Expected Learner Actions<br>☐ Review handoff history<br>☐ Zero monitoring devices<br>☐ Perform MARCH<br>☐ Verbalize initial concerns of Na too low- Bolus 150ml/hr of 3% NaCl	Modifiers<br>- Watch for Zero of devices and ensure all VS at Parameters	Participants receive handoff from treating team.<br><br>Participants may display the CPG goals for TBI<br><br>Participants may ask for VBG<br><br>ETCO2 goal: 35-40<br>CPP goal: 60-80<br>ICP goal <20<br>Na goal: 155-160
**2. Increased ICP (11 minutes into scenario)**<br>Rhythm: Sinus<br>HR: 120<br>BP: 153/85<br>RR: 18<br>O2SAT: 99%<br>T: 102°F<br>GCS: 7T<br>MAP: 107<br>ICP: 23<br>CPP: 84	Pt slowly with increasing movements and appears to be undersedated (verbal prompt from cadre during patient assessment)	Expected Learner Actions<br>☐ Recognize pt undersedated<br>☐ Verbalize concern for increased MAP and ICP<br>☐ Appropriately sedate patient (can use Ketamine or Propofol or any other medication in allowance standard with appropriate dose)	Modifiers<br>If asked- "Patient is moving and appears to be waking"	Patient is experiencing agitation due to stressors of flight leading to agitation, elevated BP and elevated ICP<br><br>Team must Recognize ↑ICP and perform an intervention<br><br>ICP Goal: <20<br>CPP goal: 60-80
**3. ICP worsens (15 minutes into scenario)**<br>Rhythm: Sinus<br>HR: 60<br>BP: 175/95<br>RR: 18<br>O2SAT: 92%<br>T: 102°F<br>GCS: 7T<br>MAP: 121<br>ICP: 27<br>CPP: 94	Patient is experiencing agitation due to stressors of flight leading to agitation, elevated BP and elevated ICP	Expected Learner Actions<br>☐ Recognize pt undersedated<br>☐ Verbalize concern for increased MAP and ICP<br>☐ Appropriately sedate patient/discuss options for lowering ICP	Modifiers<br>If asked- "Patient is moving and appears to be waking", eyes are opening	ICP Goal: <20<br>CPP goal: 60-80
**4. ICP treated**<br>Rhythm: Sinus<br>HR: 104<br>BP: 128/66<br>RR: 18<br>O2SAT: 99%<br>T: 102°F<br>GCS: 7T<br>MAP: 86<br>ICP: 18<br>CPP: 68	State occurs if any of the following actions taken after increased ICP condition occurs:<br>- Sedative or analgesic administered	Expected Learner Actions<br>☐ Recognize pt was previously undersedated<br>☐ Re-assess GCS	Modifiers<br>If asked- "Pt is GCS 3T"	Notes:
**5. Hypoventilation**<br>Rhythm: Sinus<br>HR: 104<br>BP: 128/66<br>RR: 18<br>O2SAT: 99%	Pt intubated and sedated, GCS 3T. ICP monitor in place, head wrapped.	Expected Learner Actions<br>☐ Verbalize ETCO2 to high- Adjust settings to CPG parameters<br><br>☐ inc RR/TV	Triggers<br>ETCO2 goal: 35-40	Triggers<br>ETCO2 goal: 35-40

T: 102°F<br>GCS: 7T<br>MAP: 86<br>ICP: 18<br>CPP: 68<br>**ETCO2 47**				
**6. Hypoventilation treated**<br>Rhythm: Sinus<br>HR: 104<br>BP: 128/66<br>RR: 18<br>O2SAT: 99%<br>T: 102°F<br>GCS: 7T<br>MAP: 86<br>ICP: 18<br>CPP: 68<br>**ETCO2 38**	State occurs if any of the following actions taken: Increase RR or Adjust Tidal Volume on ventilator settings	<u>Expected Learner Actions</u><br>☐ Re-assess respiratory status		
**7. Fever**<br>Rhythm: Sinus<br>HR: 104<br>BP: 128/66<br>RR: 18<br>O2SAT: 99%<br>T: 102°F<br>GCS: 7T<br>MAP: 86<br>ICP: 18<br>CPP: 68	Pt ICP stabilized, now requires fever control	<u>Expected Learner Actions</u><br>☐ Treat 102 fever with Acetaminophen 1000mg IV<br><br>☐ Lowering temperature of aircraft<br><br>☐Removing blankets if applicable	<u>Triggers</u><br>If asked, pt appears to be sweating<br><br>If Acetaminophen 1000mg IV given, return temp to 98.8 F	<u>Notes:</u>
**8. Fever treated**<br>Rhythm: Sinus<br>HR: 104<br>BP: 128/66<br>RR: 18<br>O2SAT: 99%<br>T: 98.8°F<br>GCS: 7T<br>MAP: 86<br>ICP: 18<br>CPP: 68	State occurs if any of the following actions taken:<br><br>Acetaminophen administered<br><br>Aircraft temperature lowered<br><br>Blankets removed	<u>Expected Learner Actions</u><br>☐ Re-assess temperature	<u>Modifiers</u>	<u>Notes:</u>
**9. Hyponatremia**<br>Rhythm: Sinus<br>HR: 104<br>BP: 128/66<br>RR: 18<br>O2SAT: 99%<br>T: 98.8°F<br>GCS: 7T<br>MAP: 86<br>ICP: 18<br>CPP: 68	Pt intubated and sedated, GCS 3T. ICP monitor in place, head wrapped. Initial Labs indicate	<u>Expected Learner Actions</u><br>☐ 3% hypertonic saline dose increased to 150mL/hr	<u>Modifiers</u><br>Na goal: 155-160<br><br>Participants will ask for a repeat iStat chemistry and vbg	<u>Notes:</u><br>Na: **145**<br>K: 3.9<br>Ca: 1.1<br>GLu: 162<br>Hct: 33<br>Hgb: 11<br><br>pH: 7.32<br>PCo2: 42<br>PO2: 160<br>HCo3: 18<br>BE: -3
**10. Hyponatremia treated**<br>Rhythm: Sinus<br>HR: 104<br>BP: 128/66<br>RR: 18<br>O2SAT: 99%<br>T: 98.8°F<br>GCS: 7T<br>MAP: 86<br>ICP: 18<br>CPP: 68	State occurs if any of the following actions taken: 3% hypertonic saline dose increased	<u>Expected Learner Actions</u><br>☐ Re-assess iStat values	<u>Modifiers</u>	<u>Notes:</u><br>Na: **156**<br>K: 3.7<br>Ca: 1.1<br>GLu: 167<br>Hct: 34<br>Hgb: 11<br><br>pH: 7.33<br>PCo2: 42<br>PO2: 155<br>HCo3: 19<br>BE: -2

**11. Additional possible actions from team with no changes to vital signs**	Pt stabilized. Now requires seizure prophylaxis, antibiotics redosing, and review of CPG to ensure pt meeting all targets	Expected Learner Actions<br>Redose overdue antibiotics- Cefazolin 2g<br><br>Begin seizure prophylaxis- Keppra 500mg IV<br><br>Review all CPG goals are in range	Modifiers	Notes:
**5. End of Simulation**			End after 30 total minutes	

Brian Grover

Clinical Summary (Blast resuscitation)

Scenario States, Modifiers and Triggers				
Patient State/Vitals	Patient Status	Learner Actions, Modifiers and Triggers to Move to Next State		Facilitator Notes
**1. Baseline State**<br>Rhythm: Sinus<br>HR: 125<br>BP: 90/55<br>RR: 16<br>O2SAT: 95%<br>T: 98.7° F<br>GCS: 3T<br>MAP: 66.55<br>ETCO2: 42	Pt intubated and sedated, GCS 3T.<br><br>TAC in place, no active bleeding<br><br>Pt is having signs of underresuscitation and hemorrhagic shock	Expected Learner Actions<br>☐ Review handoff history<br>☐ Zero monitoring devices<br>☐ Perform MARCH<br>☐ Identify and verbalize ongoing blast resus<br>☐ Identify pt in hemorrhagic shock and transfuse blood	Modifiers<br>- Watch for Zero of devices and ensure all VS at Parameters	Notes:<br>Participants receive handoff from treating team.<br><br>Participants may display the CPG goals for hemorrhagic shock<br><br>Participants may ask for VBG and iStat chemistry
**2. No Blood Given (10 minutes into scenario)**<br>Rhythm: Sinus<br>HR: 140<br>BP: 70/30<br>RR: 24<br>O2SAT: 88%<br>T: 98.7° F<br>GCS: 6T<br>MAP: 43.2<br>ETCO2: 36	Pt severely underresuscitated and experiencing severe hemorrhagic shock	Expected Learner Actions<br>☐ Identify pt in hemorrhagic shock and transfuse 2 units of whole blood	Modifiers<br>If asked, blood bag that was hanging at handoff is empty	Notes:<br>iStat:<br>Na: 140<br>K: 3.6<br>Ca: 0.7<br>Glu: 165<br>Hct: 21<br>Hgb: 7<br><br>pH: 7.31<br>PCo2: 38<br>PO2: 99<br>HCO3: 16<br>BE: -9
**3. 1 Unit Whole Blood Given**<br>Rhythm: Sinus<br>HR: 115<br>BP: 98/59<br>RR: 18<br>O2SAT: 93%<br>T: 98.7°F<br>GCS: 3T<br>MAP: 71.87<br>ETCO2: 36	Pt in hemorrhagic shock, slightly improving	Expected Learner Actions<br>☐ Recognize pt VS improved after 1u<br><br>☐ Verbalize continued concern for hypotension (Goal SBP 100-110)<br><br>☐ Recheck iStat CHem/VBG after 1u WB	Modifiers<br><br>If Calcium 1gram IV is given with 1st unit of WB:<br>Ca increases to 1.1	Notes:<br>VBG/iStat after 1u WB:<br>iStat:<br>Na: 140<br>K: 3.6<br>Ca: 0.7<br>Glu: 165<br>Hct: 23.7<br>Hgb: 7.9<br><br>pH: 7.33<br>PCo2: 42<br>PO2: 99<br>HCO3: 21<br>BE: -3
**4. 2nd unit of WB Given**<br>Rhythm: Sinus<br>HR: 105<br>BP: 108/65<br>RR: 18<br>O2SAT: 95%<br>T: 98.7°F<br>GCS: 3T<br>MAP: 79<br>ETCo2: 36	Pt continues to improve from hemorrhagic shock	Expected Learner Actions<br>☐ Recognize pt SBP and MAP at goal per CPG<br><br>☐ Confirm Ca 1gram IV given	Modifiers<br>VBG/iStat after 2nd unit WB:	Notes:<br>Na: 140<br>K: 3.6<br>Ca: 1.1<br>Glu: 165<br>Hct: 28.5<br>Hgb: 9.5<br>pH: 7.39<br>PCo2: 42<br>PO2: 99<br>HCO3: 22<br>BE: -3
**5. Pt understated (20mins into scenario, only after WB given)**<br>Rhythm: Sinus<br>HR: 120<br>BP: 130/76	Patient is experiencing agitation due to stressors of flight leading to agitation	Expected Learner Actions<br>☐ Recognize pt undersedated<br><br>☐ Appropriately sedate patient	Triggers<br><br>If asked "Patient is moving and appears to be waking"	Notes:<br>Participants may ask for VBG after intervention

RR: 22<br>O2SAT: 95%<br>T: 98.7°F<br>GCS: 6T<br>MAP: 81.85<br>ETCO2: 32				
**6. Sedation Given**<br>Rhythm: Sinus<br>HR: 103<br>BP: 112/67<br>RR: 18<br>O2SAT: 95%<br>T: 98.7°F<br>GCS: 3T<br>MAP: 81.85<br>ETCO2: 38	State occurs if any of the following actions taken:<br><br>Appropriately dosed with pain or sedation medications (ketamine or fentanyl drips increased or bolus)	<u>Expected Learner Actions</u><br>☐ Recognize pt is now well sedated and all parameters are at goal	<u>Modifiers</u>	<u>Notes:</u><br>iStat/VBG remain unchanged
**7. Additional possible actions from team with no changes to vital signs**	Pt stabilized. Now requires redosing, and review of CPG to ensure pt meeting all targets	<u>Expected Learner Actions</u><br>Redose overdue antibiotics-Cefazolin 1 gram IV q8hrs<br><br>Metronidazole 500mg IV q8hrs<br><br>Review all CPG goals are in range	<u>Modifiers</u>	<u>Notes:</u>
**8. End of Simulation**			<u>End after 30 total minutes</u>	

Molly Bailey

Clinical Summary (Burn and Inhalational Injury)

Scenario States, Modifiers and Triggers				
Patient State/Vitals	Patient Status	Learner Actions, Modifiers and Triggers to Move to Next State		Facilitator Notes
**1. Baseline State**<br>Rhythm: Sinus<br>HR: 132<br>BP: 70/45<br>RR: 23<br>O2SAT: 94%<br>T: 99° F<br>GCS: 3T<br>MAP: 53.25<br>ETCO2: 41	Pt intubated and sedated, GCS 3T, hypotensive, ongoing burn resuscitation<br><br>40% TBSA and inhalational injury.	Expected Learner Actions<br>☐ Review handoff history<br>☐ Zero monitoring devices<br>☐ Perform MARCH<br>☐ Identify and verbalize inhalational injury and ongoing burn resuscitation<br>☐ Identify low UOP and treat hypotension:<br>Increase LR Rate by 20-25%<br>Increase Norepinephrine drip	Modifiers<br>- Watch for Zero of devices and ensure all VS at Parameters<br><br>- If asked, UOP is 25mL/hr (goal >30mL/hr)	Notes:<br>- CVP not available<br>- Pt UOP is 25mL/hr
**2. Hypotension Treated**<br>Rhythm: Sinus<br>HR: 122<br>BP: 85/52<br>RR: 24<br>O2SAT: 93%<br>T: 99° F<br>GCS: 3T<br>MAP: 62.89<br>ETCO2: 41	Pt intubated and sedated, GCS 3T.<br><br>40% TBSA and inhalational injury	Expected Learner Actions<br><br>☐ Reassess BP after Norepinephrine drip increased	Modifiers	Notes:<br>iStat:<br>Na: 37<br>K: 3.8<br>Ca: 1.2<br>Glu: 171<br>Hct: 38<br>Hgb: 13<br><br>pH: 7.32<br>PCo2: 38<br>PO2: 71<br>HCO3: 21<br>BE: -4
**3. Hypoxia (12 mins into scenario)**<br>Rhythm: Sinus<br>HR: 126<br>BP: 92/60<br>RR: 28<br>O2SAT: 86%<br>T: 99° F<br>GCS: 3T<br>MAP: 70.56<br>ETCO2: 41	Pt in hemorrhagic shock, slightly improving	Expected Learner Actions<br>☐ Recognize pt acutely hypoxic<br>☐ Verbalize possible causes of acute hypoxia and acute interventions<br>☐ Identify high PIP alarms and consider causes<br>☐ Increase FiO2 on Ventilator<br>☐ Suction ETT	Modifiers	Notes:<br>Increased PIP on test lung to simulate high PIP alarms
**4. Increase FiO2 to 100%**<br>Rhythm: Sinus<br>HR: 126<br>BP: 92/60<br>RR: 28<br>O2SAT: 88%<br>T: 99° F<br>GCS: 3T<br>MAP: 70.56<br>ETCO2: 41	Pt hypoxia slightly improved	Expected Learner Actions<br>☐ Reassess oxygenation	Modifiers	
**5. ETT suction performed**<br>Rhythm: Sinus<br>HR: 126<br>BP: 92/60<br>RR: 28<br>O2SAT: 93%<br>T: 99° F<br>GCS: 3T<br>MAP: 70.56<br>ETCO2: 41	Pt with release of copious secretions and improvement of oxygen saturation	Expected Learner Actions<br>☐ Recognize pt need for heparin nebs to thin secretions<br><br>☐ Recognize high PIP alarms due to thick secretions	Triggers<br>If asked, patient had thick mucus plug	Notes:<br>Remove Band on test lung
**8. End of Simulation**			End after 30 total minutes	

All Data are Simulated Patient Scenarios

Stan Kim

Clinical Summary (Sepsis)

**Scenario States, Modifiers and Triggers**				
Patient State/Vitals	Patient Status	Learner Actions, Modifiers and Triggers to Move to Next State		Facilitator Notes
**1. Baseline State**<br>Rhythm: Sinus<br>HR: 90<br>BP: 113/64<br>RR: 16<br>O2SAT: 99%<br>T: 101° F<br>GCS: 3T<br>MAP: 80<br>ETCO2: 43	Pt intubated and sedated, GCS 3T, known bladder injury, vascular injury, fever, possible infection developing	Expected Learner Actions<br>☐ Review handoff history<br>☐ Zero monitoring devices<br>☐ Perform MARCH<br>☐ Identify and verbalize condition as critical and possible infection	Modifiers<br>- Watch for Zero of devices and ensure all VS at Parameters	Notes:
**2. PEEP Leak, hypoxia event (10 mins into scenario)**<br>Rhythm: Sinus<br>HR: 90<br>BP: 113/64<br>RR: 16<br>O2SAT: 91%<br>T: 101° F<br>GCS: 3T<br>MAP: 80<br>ETCO2: 43	Pt intubated and sedated, GCS 3T. Low PEEP/Low TV alarm, O2 Saturation drops out of range	Expected Learner Actions<br>☐ Identify alarm and drop in O2 saturation<br>☐ Increase FiO2 to 100% while trouble shooting alarm<br>☐ Work through cause of low PEEP/Low TV alarm and identify a hole in the ventilator circuit<br>☐ Replace Ventilator circuit	Modifiers<br>If asked, "Low PEEP or Low TV or TV not delivered alarm sounds on ventilator"<br><br>If asked, pt having minimal secretions	Notes:
**3. Hypoxia addressed**<br>Rhythm: Sinus<br>HR: 90<br>BP: 113/64<br>RR: 16<br>O2SAT: 98%<br>T: 101° F<br>GCS: 3T<br>MAP: 80<br>ETCo2: 43	State reached when:<br>Ventilator circuit correctly replaced	Expected Learner Actions<br>☐ Ensure all parameters are at goal	Modifiers	Notes:
**4. Hypotension (15 mins into scenario)**<br>Rhythm: Sinus<br>HR: 105<br>BP: 105/61<br>RR: 16<br>O2SAT: 98%<br>T: 101° F<br>GCS: 3T<br>MAP: 76<br>ETCO2: 43	Pt BP begins to decrease	Expected Learner Actions<br>☐ Recognize early hypotension	Modifiers<br>If asked, pt's dressings are dry, no active bleeding is noted<br><br>VS do not change if IV Fluid bolus given	Notes:
**5. Hypotension worsens- Septic Shock state (19 mins into scenario)**<br>Rhythm: Sinus<br>HR: 115<br>BP: 88/55<br>RR: 22<br>O2SAT: 94%<br>T: 101° F<br>GCS: 3T<br>MAP: 66<br>ETCO2: 43	Pt with worsening hypotension and tachycardia	Expected Learner Actions<br>☐ Recognize sepsis/septic shock physiology<br><br>☐ Can give IV Fluid bolus<br><br>☐ Treat with pressor: Initiate Norepinephrine drip<br><br>☐ Can give push-dose pressor while preparing Norepinephrine drip	Modifiers	Notes:<br>VS do not change until pressor drip initiated<br><br>VS do not improve if IV Fluid bolus given
**6. Worsening septic shock (23 mins into scenario- ONLY if no pressor given in**	Pt progresses into severe septic shock if no pressor is given to improve	Expected Learner Actions<br>☐ Recognize worsening sepsis/septic shock physiology	Modifiers	Notes

**previous state)**<br>Rhythm: Sinus<br>HR: 124<br>BP: 72/50<br>RR: 26<br>O2SAT: 86%<br>T: 101° F<br>GCS: 3T<br>MAP: 57<br>ETCO2: 43	hypotension. Hypoxia also developing	☐ Treat with pressor: Initiate<br>Norepinephrine drip<br><br>☐ Can give push-dose pressor while preparing<br>Norepinephrine drip		
**7. Septic Shock Treated**<br>Rhythm: Sinus<br>HR: 106<br>BP: 95/62<br>RR: 18<br>O2SAT: 93%<br>T: 101° F<br>GCS: 3T<br>MAP: 73<br>ETCO2: 43	State reached if Norepinephrine drip started	Expected Learner Actions<br>☐ Recognize improvement in hypotension and re-evaluate other parameters	Modifiers	Notes
**8. Fever treated, Pt stabilized**<br>Rhythm: Sinus<br>HR: 106<br>BP: 95/62<br>RR: 18<br>O2SAT: 93%<br>T: 99° F<br>GCS: 3T<br>MAP: 73<br>ETCO2: 43	State reached after hypotension addressed and fever treated with Acetaminophen 1gm IV	Expected Learner Actions<br>☐ Recognize improvement in hypotension and temperature; re-evaluate other parameters	Modifiers	Notes
**9. Additional actions from team with no changes to vital signs**	Pt stabilized. Now patient requires redosing and expansion of antibiotics, and review of CPG to ensure pt meeting all targets	Expected Learner Actions<br>Re-dose Ancef 1gm<br><br>Add antibiotics—broaden sepsis coverage due to Septic shock<br><br>VTE Ppx- Lovenox 40mg SC<br>Review all CPG goals are in range	Modifiers	Notes
**8. End of Simulation**			End after 30 total minutes	

George Smith

Clinical Summary (Pulmonary Embolism)

Scenario States, Modifiers and Triggers				
Patient State/Vitals	Patient Status	Learner Actions, Modifiers and Triggers to Move to Next State		Facilitator Notes
**1. Baseline State**<br>Rhythm: Sinus<br>HR: 104<br>BP: 152/73<br>RR: 18<br>O2SAT: 96%<br>T: 98° F<br>GCS: 15	Pt GCS 15, not intubated, ambulatory. Known PE, has been stable off Vent. Needs Heparin dose.	Expected Learner Actions<br>☐ Review handoff history<br>☐ Perform MARCH<br>☐ Identify and verbalize condition stable<br>☐ Identify that pt's heparin bag is empty and patient requires continued heparin treatment for PE	Modifiers<br>- Pt talking, cooperative	Notes:<br>Initial state—pt is stable, just need heparin to be re-started
**2. Hypoxia (9minutes into scenario)**<br>Rhythm: Sinus<br>HR: 120<br>BP: 140/80<br>RR: 26<br>O2SAT: 88%<br>T: 98° F	LRP is tachypneic and alerts team that they feel short of breath	Expected Learner Actions<br>☐ Identify patient is complaining of shortness of breath<br>☐ Provide supplemental oxygen via NC or face mask	Modifiers<br>If asked, patient appears stable but has complaint of dyspnea	Notes:
**3. Hypoxia addressed- Oxygen given**<br>Rhythm: Sinus<br>HR: 120<br>BP: 140/80<br>RR: 26<br>O2SAT: 94%<br>T: 98° F	State reached when:<br>Patient is given supplemental oxygen via NC or face mask	Expected Learner Actions<br>☐ Ensure pt has oxygen delivery system in place correctly and Oxygen saturation is improved	Modifiers	Notes:
**4. Pain episode (17 mins into scenario)**<br>Rhythm: Sinus<br>HR: 120<br>BP: 160/90<br>RR: 23<br>O2SAT: 94%<br>T: 98° F	LRP waves down provider and complains of 10/10 chest pain	Expected Learner Actions<br>☐ Recognize patient pain episode and continued dyspnea with oxygen requirement<br><br>☐ Treat with appropriate medication for pain	Modifiers<br>If asked, pt's pain is severe, 10/10	Notes:<br>No changes to other parameters<br>iStat:<br><br>Na: 137<br>K: 3.8<br>Ca: 1.2<br>Glu: 161<br>Hct: 30<br>Hgb: 10<br><br>pH: 7.33<br>PCO2: 94<br>HCO3: 22<br>BE: -1
**5. Pain worsens (25 mins into scenario)**<br>Rhythm: Sinus<br>HR: 120<br>BP: 160/90<br>RR: 23<br>O2SAT: 94%<br>T: 98° F	LRP complains that pain is worsening, is becoming restless due to pain	Expected Learner Actions<br>☐ Recognize patient pain episode and continued dyspnea with oxygen requirement<br><br>☐ Treat with appropriate medication for pain	Modifiers	Notes:<br>No changes to other parameters
**6. Pain treated, patient stabilized**<br>Rhythm: Sinus<br>HR: 110<br>BP: 145/82<br>RR: 20<br>O2SAT: 94%<br>T: 98° F	State reached when:<br>Pain medication given<br><br>Heparin drip restarted<br><br>Supplemental Oxygen is given	Expected Learner Actions<br>☐ Recognize patient was having hypoxia and pain due to need to heparin<br><br>☐ Ensure patient comfortable and all other parameters at goal	Modifiers	Notes

**7. Additional actions from team with no changes to vital signs**	Pt stabilized. Now patient requires review of CPG to ensure pt meeting all targets	<u>Expected Learner Actions</u><br>Ensure Heparin treatment is not interrupted<br><br>Review all CPG goals are in range		
**8. End of Simulation**			<u>End after 30 total minutes</u>	

Supplemental Material: Phase 2, Sim B Patient Scenarios for Participants

Sam Franklin // DOB: 30 May 2000 // Allergy: PCN **Current Day: JD100**

Patient History:
Pt is a 24 y/o M that presented to Role 3 ED via 9-line from the field s/p dismounted vehicle-born IED on JD098. Pt arrived intubated. Injuries include: 1) Right parietal intraparenchymal hemorrhage and 2) right parietal covexity epidural hematoma. Pt went to OR (1345z/0550z) for hematoma evacuation and Right frontal ventriculostomy. EBL 30mL. Pt transferred to the ICU postop and is stable. Best GCS was 6 and patient remains intubated.

Neurosurgery recommendations are 1) to drain Ventriculostomy for ICP > 20 mmHg with max drainage of 10cc/hr, 2) not cleared for DVT prophylaxis, 3) give seizure prophylaxis for 1 week, 4) follow ERCC severe head injury algorithms. Over last 6 hours, ICP has had intermittent increased up to 24 mmHg. EVD last drained 2 hours ago and ICP in goal since that time. Will travel HOB elevated to decrease ICP as well as receive 3% sodium solution IV and seizure prophylaxis per protocol. Will not need altitude restrictions. Pt also has C-collar for transport.

Drug allergies: Penicillin (anaphylaxis) | **Height** 67 inches | **Weight** 85 kg

Temp 98.6°F; **BP** 95/60; **MAP** 72; **HR** 115; **RR** 23; **SpO2** 96%, **ETCO2** 39; **ICP** 19;

Labs: (2 hours ago): WBC 13; Hgb 11.1; Hct 33.5; PLT 192K; Na 152; PT 14.9; INR 1.1
pH 7.33; **pCO2** 38; **pO2** 95; **HCO3** 22; **BE** -3

Rads: CT of the head revealed a complex, minimally displaced right parietal fx w/ extension into the mastoid air cells, a 1.5cm right posterior temporal contusion, right sided 3cm AP x 8mm lateral epidural hematoma. Post op CT revelead placement w/i the right foramen of Munroe and no change in the size of her intracranial hematomas.
CT of chest/abd/pelvis: negative for injury.
CXR (1 day ago): ETT in good position.

Ventilation mode: AC **FiO2** 40, **TV** 466, **Rate** 20, **PEEP** 5, **PIP** 5
No changes in the past 12 hours. Minimal secretions. 7.5 ETT, 23 at teeth.

Access: Triple Lumen Catheter (TLC) (Right Subclavian); A-Line (Right Arm)

Drips: Propofol 30mcg/kg/min; Fentanyl 100mcg/hr; 3% NS 50 cc/hr

Scheduled Medications: Keppra 500mg q12h (last dose **18h ago**); Vancomycin 1 gm Q 8h (last dose **10 h ago**); Protonix 40 mg IV Q24h (last dose 18 hours ago)

Drainage: Indwelling Catheter (Bladder) Gravity; Gastric Tube (Oral) Intermittent; (Right Extraventricular Drain) Gravity goal of 10-20 ml/hr

Diet: NPO-Nothing By Mouth

James Smith // 3 Apr 2003 // NKDA **Current Day: JD100**

Patient History:
21 y/o M/AD/USA/E-6. Downrange HX: Servicemember brought to the Role 3 s/p GSW on JD092 through spleen, stomach, transverse colon. On JD093, underwent Splenectomy, Stomach Repair, Colon Resection, Transverse Colostomy. Splenectomy vaccines given. Patient became septic with midline wound infection/dehiscence. Received large volume resuscitation and sepsis treatment in ICU and clinically improved.

On JD098: Abdominal washout and fascial closure. Vac placement to laparotomy wounds and placed to suction at -125 mmHg.

UPDATE JD099: pt had deterioration of respiratory status with increasing bilateral infiltrates, suggestive of ARDS. Blood pressure worsening and requiring levophed. Pt on AC/VC FiO2 50%. BP 90/47 HR 112 RR 35, remains sedated. 24 hr intake/output is 1.1/0.8.

Drug allergies: NKDA | Height 66 inches | Weight 90 kg

Temp 98.6°F; **BP** 80/47 (MAP 58); **HR** 112; **RR** 16; **SpO2** 92% **ETCO2** 45

Labs: **Hgb** 9; **Hct** 27; **WBC** 18
NA 135, K 4.2, CL 103, CO2 19, BUN 24, CR 1.4, GLUCOSE 142;
pH 7.36; **pO2** 65; **pCO2** 45; **HCO3** 21; **BE** -2

Rads: CT head and c-spine: negative for injury. Repeat CT chest/abd/pelvis on JD099: resolving intraabdominal fluid collection.
CXR: ETT in good position. Bilateral, patchy alveolar opacities with early consolidations. No effusion or pulmonary edema.

Ventilation mode: AC/VC, **FiO2** 50, **TV** 620, **Rate** 16, **PEEP** 8 **Pplat** 32
ETT 7.5, 23 at teeth. Thick secretions. Requiring albuterol nebs. Last given 6 hours ago.

Access: Triple Lumen Catheter (TLC) (Internal Jugular); A-Line (Right Arm)

Drips: Propofol 30mcg/kg/min; Fentanyl 100mcg/hr; Levophed 5 mcg/min

Scheduled Medications: Meropenem 1 g q8h (last dose **10h ago**); Protonix 40 mg IV Q24h (last dose 18 hours ago); Lovenox 30 mg SubQ q12h (last dose **18h ago**)

Drainage: Wound Vac (abd) Continous; Indwelling Catheter (Bladder) Gravity; ETT; Gastric Tube (Gastrium)

PMI Data: Backrest, Litter Pad; Cardiac Monitor, Pulse Ox, Pump, Suction, Ventilator, Wound Vac

Brittany Johnson // DOB: 14 Feb 1997 // NKDA **Current Day: JD100**

Patient History
Pt is a 27 yo female that presented to Role 3 on JD099 s/p VBIED. Injuries include: 1) Grade IV liver laceration; 2) Pneumothorax with Right 9 and 10 rib fxs-- s/p chest tube insertion; 3) grade III right kidney laceration. Member went to OR on JD099 for exploratory laparotomy. Grade IV R lobe liver injury involving segments 5,6,7,8. Hemostasis was achieved, but resuscitation limited by blood product shortage on site. Temporary abdominal vac closure was then placed. Pt transferred to ICU postop intubated. Total resus included: 10pRBC,10u FFP, 2u Plts, 2u Cryo. Current H/H is: 7.1/21. Minimal output from chest tube (10 cc) and temporary abdominal closure wound vac (80 cc) over last 12 hours. Last 6 hours: given 1 pRBC. Per trauma surgeon: Not cleared for DVT ppx.

Drug allergies: NKDA | Height 62 inches | Weight 65 kg

Temp 98.7°F **BP** 93/62 **HR** 125 **MAP** 72 **RR** 16 **SpO2** 95%

Labs: WBC 15 **Hgb** 7.1 **Hct** 21 **PLT** 161
NA 135, K 3.7, CL 103, CO2 18, BUN 27, CR 1.5, GLUCOSE 182;
pH 7.35 **pCO2** 42 **pO2** 99 **HCO3** 18 **BE** -6 **Ca** 0.8; Lactate 3.5

Rads: CT head: normal. CT chest/abd/pelvis: Chest- mod right pneumothorax w/ small pulmonary contusion, right rib 9/10 fracture; Abd/Pelv Grade IV liver laceration; grade III right kidney laceration.
CXR (6 hours ago): ETT in good position. R chest tube in good position. Minimal R pneumothorax remaining.

Ventilation Mode: AC **FiO2** 30% **TV** 350 **Rate** 18 **PEEP** 5 **ETCO2** 32 **Pplat** 15
No changes in the past 12 hours. Minimal secretions. 7.5 ETT, 23 at teeth.
Chest tube placed connected to Pleur-evac on -10 suction. Tidaling present. No air leak.

Access: Triple Lumen Catheter (TLC) (Right Subclavian); A-Line (Right Arm); Saline Lock (Left Arm)

Drips: Propofol 30mcg/kg/min; Fentanyl 100mcg/hr

Scheduled Medications: Cefazolin 1 g q8h (last dose **10h ago**); Flagyl 500 mg IV q6h (last dose **8hrs ago**) Protonix 40 mg IV Q24h (last dose 18 hours ago)

Drainage: Indwelling Catheter (Bladder) Gravity; Gastric Tube (Oral) Intermittent; Pleur-evac (Right chest) water seal; Wound Vac (abdomen)

PMI Data: Backrest; Litter Pad; Cardiac Monitor; Pulse Ox; IV Pump; Suction; Ventilator; Wound Vac

Other Equipment: Pleur-evac

Diet: NPO-Nothing By Mouth

Eric Daniels // DOB: 4 JUN 1998 // NKDA **Current Day: JD100**

Pt History: Pt is a 26 y/o AD USA male that presented to Role 3 on JD 098 s/p injury to bilateral lower extremities. Mechanism of injury was crush to bilateral legs from vehicle. GCS 14 on admit. CT traumogram performed, RESULTS: status post bilateral LE fasciotomy with extensive soft tissue injury, bilateral tib/fib fractures. The patient is status post bilateral tib/fib ORIF. Pt went to OR on JD099 1300Z/1945Z.
OP NOTES: Prophylactic four compartment fasciotomy to bilateral lower legs. Wet to dry dressings placed bilaterally. Pt transferred to ICU postop stabilized. Distal pulses intact postoperatively. Pt stable on ventilator. Total resus included: FFP x 6 U, RBCs x 13 U, Platelets x 2 U, whole blood x 1 U. Initial hemoglobin was 7.4 at 1300 Z, JD 099. Recheck of Hgb as of 2030Z at 9.7/29. Pt has increasing AKI, creatinine 2.4. Dialysis unavailable on site. Hyperkalemic to 6.0. Calcium gluconate, lasix and bicarb given 12 hours ago. Urine output 50 ml/hr. I/O over 24 hours: 2.4L in/1.1 L out.

Drug allergies: NKDA **|** **Height** 69 inches | **Weight** 75 kg

Temp 98.0°F; **BP** 152/73; **HR** 90; **RR** 18; **SpO2** 96%, Vent, **ETCO2** 40

Labs (4 hours ago): WBC 8,000 **Hgb** 9.7 **Hct** 29 **PLT** 125
NA 136, **K** 5.2, **CL** 106, **CO2** 21, **BUN** 31, **CR** 2.4, **GLU** 107;
pH 7.36 **pCO2** 41 **pO2** 99 **HCO3** 21 **BE** -2

Rads: CT head: negative. CT chest/abd/pelvis: negative. CT Lower extremities: see report above.
CXR: ETT in good position. Lungs clear.

Ventilation mode: AC, **FiO2** 30% **TV** 424 **Rate** 18, **PEEP** 5 **Pplat** 15
No changes in the past 12 hours. Minimal secretions. 7.5 ETT, 23 at teeth.

Access: Triple Lumen Catheter (TLC) (Right Subclavian); A-Line (Right Arm)

Drips: Normal saline 150 ml/hr; Propofol 30mcg/kg/min; Fentanyl 100mcg/hr

Scheduled Medications: Protonix 40 mg IV Q24h (last dose 18 hours ago); Lovenox 30 mg SubQ q12h (last dose **18h ago**); Cefazolin 1 gm IV q8h (last dose **10hrs ago**)

Drainage: Indwelling Catheter (Bladder) Gravity; Gastric Tube (Oral) Intermittent

PMI Data: Backrest, Litter Pad; Cardiac Monitor, Pulse Ox, IV Pump, Suction, Ventilator

Diet: NPO-Nothing By Mouth

Jamie Williams // DOB: 23 Jan 1995 // NKDA **Current Day: JD100**

<u>This is your live role player. The patient may be male or female</u> depending on staff availability.

Patient History: 29 yo patient sustained multiple penetrating fragment wounds from a negligent discharge on JD099. Patient was in a common room when pt heard gunfire and felt pain in Lt chest and Lt leg. On arrival to Role 3, pt was A&O x 3, GCS 15/15. Left Chest tube was placed and put on suction. Blood output was 300 cc over 1st hour, total output 500 cc, 30 cc over last hour. Small L PTX present. Chest tube to suction at -20 mmHg. Atrium Express chamber in place and chest tube is tidaling. Bullet removal and washout to left lateral distal lower extremity. Dressing placed. Neurovascularly intact distally. Fast exam negative.

Drug allergies: NKDA | **Height** 77 inches | **Weight** 80kg

Temp 97.0°F; **BP** 141/75; **HR** 104; **RR** 18; **SpO2** 97% on room air

Labs: Hgb 15.8; **Hct** 50.2; **WBC** 14400; **Plt** 186
No VBG or BMP available.

Rads: Initial CT imaging: CT head and c-spine: negative. CT of chest/abdomen/pelvis showed a Lt apical pneumothorax, moderate Lt hemothorax. Otherwise negative.
Xray chest (6 hours ago): left chest tube in good position. Minimal remaining hemothorax.
Xray LLE: no fracture to LLE. No retained foreign body.

Access: Saline Lock (Left Arm); Radial arterial line (Left Arm)

Drips: LR 110ml/hr

Scheduled medications: Tylenol 1g Q8H (last dose **<u>10 hours ago</u>**); Dilaudid 0.5 mg IV q6H prn pain (last given **<u>10 hours ago</u>**); Ancef 1 g Q8H (last dose **<u>10 hours ago</u>**); Lovenox 30 mg SubQ q12H (last given **<u>18 hours ago</u>**)

Drainage: Chest Tube (Left chest) to suction

PMI Data: Backrest; Litter Pad; Cardiac Monitor; Pulse Ox; IV Pump; Suction

Diet: Regular. Cleared for oral intake and meds as needed.

Supplemental Material: Phase 2, Sim B Patient Summaries for Instructors

Sam Franklin

Clinical Summary (TBI/EVD)

Scenario States, Modifiers and Triggers				
Patient State/Vitals	Patient Status	Learner Actions, Modifiers and Triggers to Move to Next State		Facilitator Notes
**1. Baseline State: Low cerebral perfusion pressure**<br>Rhythm: Sinus<br>HR: 115<br>BP: 95/60<br>RR: 23<br>O2SAT: 96%<br>T: 99° F<br>GCS: 3T<br>ETCO2: 39<br>ICP: 19<br>MAP: 72<br>CPP: 53	Pt GCS 3T, EVD in place. C-Collar in good position. Pt with low CPP at hand-off. ICP at goal.	Expected Learner Actions<br>☐ Review handoff history<br>☐ Perform MARCH<br>☐ Identify and verbalize CPP not a goal<br>☐ Give pressor to address low CPP/low SBP<br>☐ Consider changing propofol due to hypotension	Modifiers<br>No active bleeding	Initial state—pt is hypotensive out of SBP parameters for CPG—Pressor is intervention of choice<br><br>iStat:<br>Na: 152<br>K: 3.8<br>Ca: 1.0<br>Glu: 157<br>Hct: 33<br>Hgb: 11<br><br>pH: 7.32<br>PCO2: 42<br>PO2: 160<br>HCO3: 18<br>BE: -3
**2. Partially improved cerebral perfusion pressure**<br>Rhythm: Sinus<br>HR: 108<br>BP: 104/65<br>RR: 23<br>O2SAT: 96%<br>T: 99° F<br>GCS: 3T<br>ETCO2: 39<br>ICP: 19<br>MAP: 78<br>CPP: 59	State reached following reduction in propofol sedation dose	Expected Learner Actions<br>☐ Recognize slight improvement in SBP and CPP<br><br>☐ Recognize continued need for Pressor<br>(push dose followed by Vasopressin drip as first choice)	Modifiers<br>CPP and SBP still not at goal per CPG	Notes:<br>Goals:<br>CPP: 60-70<br>SBP>110<br>ICP<22
**3. Cerebral perfusion pressure at goal**<br>Rhythm: Sinus<br>HR: 118<br>BP: 112/70<br>RR: 23<br>O2SAT: 96%<br>T: 99° F<br>GCS: 3T<br>ETCO2: 39<br>ICP: 19<br>MAP: 84<br>CPP: 65	State reached when:<br>Vasopressor administered	Expected Learner Actions<br>☐ Recognize first-line choice for pressor is Vasopressin<br><br>☐ Recognize hypotension improved following pressor initiation	Modifiers	Notes:
**4. Increased ICP (15 mins into scenario)**<br>Rhythm: Sinus<br>HR: 118<br>BP: 112/70<br>RR: 23<br>O2SAT: 99%<br>T: 99° F<br>GCS: 3T<br>ETCO2: 39<br>ICP: 27<br>MAP: 84<br>CPP: 57	Patient ICP increases at take off. EVD was last drained 2hrs prior to hand-off.	Expected Learner Actions<br>☐ Recognize ICP elevated and work through cause and treatment<br><br>☐ Drain EVD per NSGY recs	Modifiers<br>If asked, pt is well sedated, C collar in good position<br><br>NSRG recs: drain Ventriculostomy for ICP > 20 mmHg with max drainage of 10cc/hr	Notes:<br>No changes to other parameters

**5. Worsening ICP (20 mins into scenario)**<br>Rhythm: Sinus<br>HR: 60<br>BP: 175/95<br>RR: 23<br>O2SAT: 92%<br>T: 99° F<br>GCS: 3T<br>ETCO2: 39<br>ICP: 29<br>MAP: 121<br>CPP: 92	Pt with worsening ICP elevation, Experiencing intracranial hypertension	<u>Expected Learner Actions</u><br>☐ Recognize ICP elevated and work through cause and treatment<br><br>☐ Drain EVD per NSGY recs<br><br>☐ Recognize Na out of range and treat	<u>Modifiers</u><br>Occurs only if increased ICP interventions are not performed by 20 minutes	<u>Notes:</u><br>No changes to other parameters
**6. EVD Drained**<br>Rhythm: Sinus<br>HR: 60<br>BP: 175/95<br>RR: 23<br>O2SAT: 92%<br>T: 99° F<br>GCS: 3T<br>ETCO2: 39<br>ICP: 19<br>MAP: 121<br>CPP: 102	State reached when:<br>EVD drained correctly- 10cc per NSGY recs<br><br>Pt experiencing intracranial hypertension	<u>Expected Learner Actions</u><br>☐ Recognize ICP normalized but CPP still out of range<br><br>☐ Recognize Na not at goal- adjust 3% NaCl rate by increasing by 10mL/hr<br><br>☐ Paralyze as last resort to lower CPP	<u>Modifiers</u><br>Pt is well sedated<br>EVD drained appropriately<br>CPP still elevated	<u>Notes</u><br>Goals:<br>CPP: 60-70<br>SBP>110<br>ICP<22
**7. Vecuronium Given**<br>Rhythm: Sinus<br>HR: 106<br>BP: 115/72<br>RR: 18<br>O2SAT: 98%<br>T: 99° F<br>GCS: 3T<br>ETCO2: 39<br>ICP: 21<br>MAP: 84<br>CPP: 63	Pt stabilized after all interventions for intracranial hypertension given	<u>Expected Learner Actions</u><br>☐ Recognize pt stabilized following intracranial hypertension event<br><br>☐ Re-evaluate all parameters are in goal	<u>Modifiers</u>	<u>Notes</u>
**9. Additional actions from team with no changes to vital signs**	Pt stabilized after intracranial hypertension treated	<u>Expected Learner Actions</u><br>Adjust Na—Increase Rate by 10mL/hr	<u>Modifiers</u>	<u>Notes</u><br>Once 3% rate adjusted:<br>Na: 156<br>K: 3.7<br>Ca: 1.1<br>Glu: 160<br>Hct: 34<br>Hgb: 11<br><br>pH: 7.33<br>PCO2: 38<br>PO2: 155<br>HCO3: 19<br>BE: -2
**8. End of Simulation**			<u>End after 30 total minutes</u>	

James Smith
Clinical Summary (ARDS)

Scenario States, Modifiers and Triggers				
Patient State/Vitals	Patient Status	Learner Actions, Modifiers and Triggers to Move to Next State		Facilitator Notes
**1. Baseline State**<br>Rhythm: Sinus<br>HR: 112<br>BP: 80/47<br>RR: 16<br>O2SAT: 92%<br>T: 99° F<br>GCS: 3T<br>ETCO2: 45<br>ICP: 19<br>MAP: 58<br><br>Initial Vent Settings:<br>TV: 440<br>RR: 24<br>PEEP: 8<br>PIP: 28<br>FiO2: 50%	Pt GCS 3T, known ARDS and Hypotension<br><br>TV too high at handoff per ARDSnet goals.	Expected Learner Actions<br>☐ Review handoff history<br>☐ Perform MARCH<br>☐ Identify and verbalize hypotension<br><br>☐ Avoid hypoxia<br>☐ Recognize ARDS objectives<br>☐Correct hyperventilation<br><br>Correct Tidal Volume (<8cc/kg, TV <438)<br><br>Increase RR to maintain minute ventilation (9.9L)	Modifiers<br>No active bleeding	Initial state- pt is hypotensive with ARDS<br>iStat:<br>Na: 140<br>K: 3.6<br>Ca: 1.1<br>Glu: 165<br>Hct: 27<br>Hgb: 9<br><br>pH: 7.36<br>PCO2: 45<br>PO2: 65<br>HCO3: 21<br>BE: -2
**2. Norepinephrine drip increased**<br>Rhythm: Sinus<br>HR: 115<br>BP: 95/62<br>RR: 16<br>O2SAT: 92%<br>T: 99° F<br>GCS: 3T<br>ETCO2: 45<br>ICP: 19<br>MAP: 73	State reached following increase in Norepinephrine drip increased	Expected Learner Actions<br>☐ Reassess blood pressure	Modifiers<br>No change in other parameters	Notes:
**3. Hypoxia event (14 mins into scenario)**<br>Rhythm: Sinus<br>HR: 130<br>BP: 95/62<br>RR: 16<br>O2SAT: 78%<br>T: 99° F<br>GCS: 3T<br>ETCO2: 45<br>MAP: 73	Patient experiencing worsening ARDS and having acute hypoxia	Expected Learner Actions<br>☐ Recognize acute hypoxia and intervene by increasing FiO2 and/or manually bagging patient (keep ETT clamped)<br><br>☐ Should also perform inline suction, give albuterol neb, increase PEEP per ARDS Net protocol	Modifiers<br>Increased PIP noted on vent alarms	Notes:<br>Main actions are to increase FiO2 and PEEP as these are address oxygenation<br><br>Baseline Vent settings:<br>FIO2: 50%<br>PIP: 28<br>TV: 440<br>BPM: 24<br>PEEP: 8
**4. Hypoxia addressed- Increased FiO2 or manual ventilation with BVM**<br>Rhythm: Sinus<br>HR: 130<br>BP: 115/95<br>RR: 16<br>O2SAT: 86%<br>T: 99° F<br>GCS: 3T<br>ETCO2: 45<br>MAP: 73	State reached when:<br>Pt FiO2 increased to 100%<br><br>Patient's hypoxia slightly improved with high FiO2	Expected Learner Actions<br>☐ Reassess oxygenation<br><br>Still not at goal, consider increasing PEEP per ARDSNET	Modifiers	Notes:<br>No changes to other parameters
**5. Hypoxia addressed- Increase in PEEP**<br>Rhythm: Sinus<br>HR: 130<br>BP: 115/95<br>RR: 16<br>O2SAT: 92%	State reached when:<br>PEEP increased to 10<br><br>Patient's hypoxia improved with	Expected Learner Actions<br>☐ Recognize improvement in oxygenation with both FiO2 and PEEP increased per ARDSNET	Modifiers	Notes:<br>No changes to other parameters

T: 99° F<br>GCS: 3T<br>ETCO2: 45<br>MAP: 73	high FiO2/high PEEP			
**6. End of Simulation**			End after 30 total minutes	

Brittany Johnson

Clinical Summary (Abd + chest tube)

Scenario States, Modifiers and Triggers				
Patient State/Vitals	Patient Status	Learner Actions, Modifiers and Triggers to Move to Next State		Facilitator Notes
**1. Baseline State**<br>Rhythm: Sinus<br>HR: 125<br>SBP: 93/62<br>RR: 16<br>O2SAT: 95%<br>T: 98.7° F<br>ETCO2: 38<br>MAP: 72	Pt with abdominal and chest trauma, Chest tube in place. Hemorrhagic shock state, resuscitation ongoing.	Expected Learner Actions<br>☐ Review handoff history<br>☐ Perform MARCH<br>☐ Identify patient is in hemorrhagic shock and hypotensive. Transfuse blood and given Calcium 1 gram IV	Modifiers<br>- No active bleeding<br><br>- Blood bag empty at hand-off	Initial iStat:<br>Na: 138<br>K: 3.8<br>Ca: 0.9<br>Glu: 142<br>Hct: 22<br>Hgb: 7.2<br><br>pH: 7.31<br>PCO2: 32<br>PO2: 99<br>HCO3: 20<br>BE: -5
**2. Hypotension worsening- No blood given (10 mins into scenario)**<br>Rhythm: Sinus<br>HR: 131<br>SBP: 80/40<br>RR: 19<br>O2SAT: 93%<br>T: 98.7° F<br>ETCO2: 36<br>MAP: 53	Patient is experiencing worsening hypotension due to underresuscitation and requires blood transfusion	Expected Learner Actions<br>☐ Identify patient now in hemorrhagic shock and requires blood transfusion	Modifiers	Notes:
**3. 1u WB Transfused**<br>Rhythm: Sinus<br>HR: 121<br>SBP: 88/58<br>RR: 19<br>O2SAT: 94%<br>T: 98.7° F<br>ETCO2: 36<br>MAP: 68	State reached when Patient is transfused 1u WB	Expected Learner Actions<br>☐ Reassess blood pressure<br><br>☐ Recognize BP not at goal, requires additional unit of WB and Calcium 1gm IV	Modifiers	Notes:<br>AFter 1u WB only given:<br>Na: 138<br>K: 3.8<br>Ca: 0.9<br>Glu: 142<br>Hct: 27<br>Hgb: 9<br><br>pH: 7.35<br>PCO2: 41<br>PO2: 99<br>HCO3: 21<br>BE: -3
**4. 2nd unit WB transfused and Calcium given**<br>Rhythm: Sinus<br>HR: 121<br>SBP: 88/58<br>RR: 19<br>O2SAT: 94%<br>T: 98.7° F<br>ETCO2: 36<br>MAP: 68	State reached when Patient is transfused 2nd unit WB and Calcium 1gm IV	Expected Learner Actions<br>☐ Recognize SBP at goal after 2nd unit<br><br>☐ Obtain repeat iStat to confirm Calcium and acidosis improved	Modifiers	Notes:<br>**iStat:**<br>Na: 138<br>K: 3.8<br>Ca: 1.2<br>Glu: 142<br>Hct: 27<br>Hgb: 9<br><br>pH: 7.39<br>PCO2: 41<br>HCO3: 21<br>PO2: 99<br>BE: -3
**5. Additional actions from team with no changes to vital signs**	Pt stabilized. Now patient requires redosing of antibiotics	Expected Learner Actions<br>Cefazolin 1gm IV q6<br>Metronidazole 500mg IV q6		
**6. End of Simulation**			End after 30 total minutes	

All Data are Simulated Patient Scenarios

Eric Daniels

Clinical Summary (Crush)

Scenario States, Modifiers and Triggers				
Patient State/Vitals	Patient Status	Learner Actions, Modifiers and Triggers to Move to Next State		Facilitator Notes
**1. Baseline State**<br>Rhythm: Sinus<br>HR: 90<br>SBP: 152/73<br>RR: 18<br>O2SAT: 96%<br>T: 98° F<br>ETCO2: 40<br>MAP: 99	Pt with crush injury, hyperkalemia. AKI and rhabdomyolysis.	Expected Learner Actions<br>☐ Review handoff history<br>☐ Perform MARCH<br>☐ Identify patient crush injury physiology resulting in hyperkalemia and rhabdomyolysis<br>☐ Treat Hyperkalemia	Modifiers<br>No active bleeding	Peaked T waves on telemetry<br>Initial handoff labs:<br>Na: 136<br>K: 5.2<br>Cl: 106<br>CO2: 21<br>BUN: 31<br>Cr: 2.4<br>Glucose: 107<br><br>pH: 7.36<br>PCO2: 41<br>PO2: 99<br>HCO3: 21<br>BE: -2
**2. Moderate Hyperkalemia (8 mins into scenario)**<br>Rhythm: Sinus<br>HR: 70<br>SBP: 121/61<br>RR: 20<br>O2SAT: 96%<br>T: 98° F<br>ETCO2: 40<br>MAP: 81	Moderate hyperkalemia changes	Expected Learner Actions<br><br>☐ Recognize peaked T-waves and QRS widening on telemetry and give Calcium<br><br>☐ Recognize worsening hyperkalemia and treat with: Insulin, Furosemide, Albuterol to shift K intracellularly	Modifiers	Notes:<br>Na: 141<br>K: 6.5<br>Ca: 1.1<br>Glu: 154<br>Hct: 29<br>Hgb: 10<br><br>pH: 7.36<br>PCO2: 41<br>PO2: 99<br>HCO3: 21<br>BE: -2
**3. Severe Hyperkalemia (15 mins into scenario if no treatment given)**<br>Rhythm: Sinus<br>HR: 50<br>SBP: 81/50<br>RR: 24<br>O2SAT: 88%<br>T: 98° F<br>ETCO2: 35<br>MAP: 60	State reached if no Calcium or Hyperkalemia treatments given in prior state<br>Pt status is declining-hypotensive and hypoxic	Expected Learner Actions<br>☐ Recognize peaked T-waves and QRS widening on telemetry and give Calcium<br><br>☐ Recognize worsening hyperkalemia, sine wave on tele and treat with: Insulin, Furosemide, Albuterol to shift K	Modifiers	Notes:<br>Sine wave on telemetry
**4. Hyperkalemia Treated**<br>Rhythm: Sinus<br>HR: 91<br>SBP: 130/63<br>RR: 20<br>O2SAT: 94%<br>T: 98.7° F<br>ETCO2: 39<br>MAP: 85	State reached when<br>Patient is treated with Calcium AND 1 of the following: insulin, Lasix, albuterol, glucose, sodium bicarbonate to shift potassium	Expected Learner Actions<br>☐ Recognize improvement in pt status after hyperkalemia treatments given	Modifiers	Notes:<br>**Normal Sinus Rhythm on telemetry**
**5. End of Simulation**			End after 30 total minutes	

Jamie Williams

Clinical Summary (GSW)

Scenario States, Modifiers and Triggers				
Patient State/Vitals	Patient Status	Learner Actions, Modifiers and Triggers to Move to Next State		Facilitator Notes
**1. Baseline State**<br>Rhythm: Sinus<br>HR: 104<br>SBP: 141/75<br>RR: 18<br>O2SAT: 97% on RA<br>T: 98° F	Pt not intubated, ambulatory GSW with chest tube in place. Currently stable. Requires re-dosage of pain medications, antibiotics and Lovenox.	<u>Expected Learner Actions</u><br>☐ Review handoff history<br>☐ Perform MARCH<br>☐ Identify overdue medications	<u>Modifiers</u><br>- CT in place at handoff, tidaling	<u>Notes</u><br>Na: 141<br>K: 3.9<br>Ca: 1.1<br>Glu: 148<br>Hct: 29<br>Hgb: 14<br><br>pH: 7.36<br>PCO2: 41<br>PO2: 95<br>HCO3: 21<br>BE: -2
**2. Pain Event (10 mins into scenario)**<br>Rhythm: Sinus<br>HR: 120<br>SBP: 160/90<br>RR: 23<br>O2SAT: 97% on RA<br>T: 98° F	Patient is stable but experiencing pain due to overdue pain medication	<u>Expected Learner Actions</u><br>☐ Recognize patient complaining of pain and treat with pain medications that are overdue	<u>Modifiers</u><br>Patient is moving around due to pain	<u>Notes:</u>
**3. Chest tube becomes disconnected (18 mins into scenario)**<br>Rhythm: Sinus<br>HR: 120<br>SBP: 135/72<br>RR: 26<br>O2SAT: 86% on RA<br>T: 98° F	Pt is tachypneic and complains of dyspnea	<u>Expected Learner Actions</u><br>☐ Recognize patient hypoxia and give supplemental oxygen to stabilize<br><br>☐ Troubleshoot causes of acute hypoxia, work through chest tube connections	<u>Modifiers</u>	<u>Notes:</u><br>Patient is repeatedly complaining of shortness of breath
**4. Supplemental Oxygen given**<br>Rhythm: Sinus<br>HR: 120<br>SBP: 135/72<br>RR: 26<br>O2SAT: 92% on RA<br>T: 98° F	State reached when<br>Patient is given supplemental oxygen via a Nasal Cannula or NRB mask	<u>Expected Learner Actions</u><br>☐ Recognize improvement in pt oxygenation after supplemental oxygen given<br><br>☐ Investigate CT connections- will find that the CT became disconnected when patient was having pain episode- reconnect CT appropriate and check for position and tidaling of CT reservoir	<u>Modifiers</u>	<u>Notes:</u><br>Patient continues to complain of dyspnea and remains tachycardic
**5. Chest Tube reconnected**<br>Rhythm: Sinus<br>HR: 109<br>SBP: 143/76<br>RR: 19<br>O2SAT: 93% on RA<br>T: 98° F	State reached only if team finds the Chest tube malfunction and reconnects the chest tube appropriately<br><br>Pt is now stable	<u>Expected Learner Actions</u><br>☐ Recognize patient is now stabilized	<u>Modifiers</u>	<u>Notes</u><br>Patient tachypnea and shortness of breath resolved
**6. Additional actions from team with no changes to vital signs**	Pt stabilized. Now patient requires redosing of antibiotics and DVT ppx	<u>Expected Learner Actions</u><br>Cefazolin 1gm IV q8<br>Lovenox 30mg SC q12<br>Acetaminophen 1g q8hr<br>Dilaudid 0.5mg IV q6hr prn		
**7. End of Simulation**			<u>End after 30 total minutes</u>	
