# Supplementary material for: Heart Rate Variability and Body Motion as Digital Biomarkers of Task Workload During Military En Route Critical Care Simulations
Source: Sensors (Basel). 2026 Jun 5;26(11):3596. doi: 10.3390/s26113596 (PMC13259423; doi:10.3390/s26113596)
Supplement: Supplementary file 1 [file sensors-26-03596-s001.zip › Supplementary materials file S2.pdf]

Supplementary materials file S2

Statistical Results

Manuscript Title: Heart Rate Variability and Body Motion as Digital Biomarkers of Task Workload During Military En Route Critical Care Simulations

B.1 Convergent Validity Analysis

Sample: N=61-72 subjects contributing 78-90 scenario observations.

B.1.1 Unadjusted Correlations: HRV Metrics and NASA-TLX

Model: Linear mixed-effects models (LMM) with random subject intercepts: `NASA_TLX ~ HRV_metric + (1|Subject)`

Sample: N=78 paired scenarios from 61 subjects.

Table B.1.1: Unadjusted Correlations Between HRV Metrics and NASA-TLX Dimensions

HRV Metric	NASA-TLX Dimension	Spearman ρ	Spearman p	LMM β	LMM 95% CI	LMM p
**RMSSD (ms)**	Overall Workload	-0.064	0.575	0.014	[-0.108, 0.135]	0.827
	Mental Demand	-0.053	0.646	0.010	[-0.097, 0.118]	0.851
	Physical Demand	-0.220	0.053	-0.094	[-0.230, 0.042]	0.175
	Temporal Demand	-0.064	0.577	-0.011	[-0.114, 0.092]	0.834
	Effort	0.046	0.686	0.049	[-0.068, 0.165]	0.415
	Frustration	0.013	0.910	0.076	[-0.062, 0.215]	0.281
	Performance	0.017	0.882	-0.004	[-0.126, 0.119]	0.953
**LF/HF Ratio**	Overall Workload	0.175	0.126	0.037	[-0.159, 0.233]	0.712
	Mental Demand	0.118	0.305	0.028	[-0.140, 0.196]	0.745
	Physical Demand	0.201	0.077	0.135	[-0.099, 0.368]	0.259
	Temporal Demand	0.205	0.071	0.088	[-0.069, 0.246]	0.272
	Effort	0.165	0.149	-0.001	[-0.198, 0.197]	0.994
	Frustration	0.023	0.844	-0.065	[-0.299, 0.168]	0.585
	Performance	0.031	0.785	0.026	[-0.176, 0.229]	0.798
**Heart Rate (bpm)**	Overall Workload	0.027	0.813	-0.002	[-0.209, 0.205]	0.982
	Mental Demand	0.001	0.992	-0.038	[-0.220, 0.144]	0.680
	Physical Demand	-0.050	0.665	0.004	[-0.241, 0.250]	0.972
	Temporal Demand	0.067	0.562	0.066	[-0.108, 0.239]	0.458
	Effort	-0.172	0.131	-0.128	[-0.330, 0.073]	0.212
	Frustration	0.023	0.845	0.009	[-0.235, 0.252]	0.944
	Performance	0.068	0.556	0.076	[-0.138, 0.290]	0.487

Note: All unadjusted HRV correlations non-significant (all LMM p > 0.05).

B.1.2 Unadjusted Correlations: Motion Metrics and NASA-TLX

Model: Linear mixed-effects models (LMM) with random subject intercepts: `NASA_TLX ~ Motion_metric + (1|Subject)`

Sample: N=90 paired scenarios from 72 subjects.

Table B.1.2: Unadjusted Correlations Between Motion Metrics and NASA-TLX Dimensions

Motion Metric	NASA-TLX Dimension	Spearman ρ	Spearman p	LMM β	LMM 95% CI	LMM p
**Acc SD (m/s²)**	Overall Workload	0.108	0.311	-0.031	[-0.243, 0.181]	0.775
	Mental Demand	0.051	0.635	-0.063	[-0.233, 0.108]	0.470
	Physical Demand	0.194	0.067	0.185	[-0.051, 0.420]	0.124
	Temporal Demand	0.052	0.625	-0.042	[-0.196, 0.113]	0.598
	Effort	0.043	0.685	-0.070	[-0.282, 0.141]	0.514
	Frustration	0.144	0.176	0.097	[-0.127, 0.321]	0.398
	Performance	-0.011	0.917	-0.068	[-0.261, 0.126]	0.492
**Acc Max (m/s²)**	Overall Workload	-0.057	0.592	-0.107	[-0.250, 0.036]	0.142
	Mental Demand	-0.092	0.391	-0.083	[-0.199, 0.033]	0.163
	Physical Demand	0.023	0.831	0.018	[-0.145, 0.180]	0.831
	Temporal Demand	-0.034	0.751	-0.055	[-0.161, 0.051]	0.309
	Effort	-0.041	0.700	-0.108	[-0.249, 0.033]	0.134
	Frustration	0.033	0.760	-0.036	[-0.191, 0.120]	0.653
	Performance	-0.031	0.773	-0.072	[-0.205, 0.061]	0.286
**Speed Mean (km/h)**	Overall Workload	0.171	0.106	0.055	[-0.136, 0.246]	0.574
	Mental Demand	0.111	0.299	0.015	[-0.138, 0.168]	0.848
	Physical Demand	0.176	0.097	0.188	[-0.026, 0.402]	0.085
	Temporal Demand	0.098	0.359	-0.015	[-0.159, 0.129]	0.842
	Effort	0.086	0.420	0.007	[-0.181, 0.196]	0.939
	Frustration	0.168	0.113	0.096	[-0.108, 0.300]	0.355
	Performance	0.052	0.625	0.028	[-0.147, 0.203]	0.751
**Speed SD (km/h)**	Overall Workload	0.119	0.266	0.013	[-0.178, 0.205]	0.891
	Mental Demand	0.081	0.448	0.001	[-0.153, 0.156]	0.986
	Physical Demand	0.199	0.061	0.180	[-0.031, 0.392]	0.095
	Temporal Demand	0.017	0.877	-0.069	[-0.217, 0.079]	0.362
	Effort	0.128	0.230	0.027	[-0.161, 0.215]	0.778
	Frustration	0.160	0.132	0.085	[-0.119, 0.289]	0.414
	Performance	0.004	0.973	-0.051	[-0.226, 0.125]	0.571
**Speed Max (km/h)**	Overall Workload	0.059	0.581	-0.072	[-0.234, 0.091]	0.388
	Mental Demand	0.015	0.890	-0.052	[-0.181, 0.077]	0.432
	Physical Demand	0.144	0.174	0.079	[-0.105, 0.263]	0.399
	Temporal Demand	-0.012	0.908	-0.095	[-0.217, 0.026]	0.124
	Effort	0.101	0.344	-0.028	[-0.187, 0.132]	0.735

	Frustration	0.097	0.365	0.027	[-0.147, 0.201]	0.758
	Performance	-0.029	0.787	-0.080	[-0.229, 0.069]	0.292
**Distance (m)**	Overall Workload	0.118	0.267	-0.020	[-0.217, 0.178]	0.845
	Mental Demand	0.080	0.455	-0.013	[-0.170, 0.143]	0.867
	Physical Demand	0.208	0.050	0.186	[-0.034, 0.406]	0.098
	Temporal Demand	0.028	0.790	-0.108	[-0.260, 0.043]	0.161
	Effort	0.109	0.307	0.022	[-0.170, 0.213]	0.825
	Frustration	0.154	0.148	0.065	[-0.144, 0.274]	0.542
	Performance	-0.003	0.977	-0.068	[-0.248, 0.112]	0.461
**Cadence Active (%)**	Overall Workload	0.118	0.269	0.017	[-0.212, 0.246]	0.886
	Mental Demand	0.068	0.525	-0.001	[-0.181, 0.179]	0.991
	Physical Demand	0.200	0.059	0.231	[-0.029, 0.490]	0.081
	Temporal Demand	0.039	0.715	-0.057	[-0.225, 0.111]	0.507
	Effort	0.088	0.411	0.035	[-0.191, 0.260]	0.762
	Frustration	0.153	0.149	0.098	[-0.147, 0.342]	0.433
	Performance	-0.025	0.817	-0.056	[-0.267, 0.155]	0.602

Note: No motion-NASA correlations reached statistical significance (all LMM p > 0.05).

B.1.3 Baseline-Adjusted Correlations: LF/HF Ratio and Heart Rate

Model: `NASA_TLX ~ Simulation_HRV + Baseline_HRV + (1|Subject)`

Sample: N=71 scenarios from 56 subjects.

Note: Baseline-adjusted RMSSD correlations reported in manuscript Table 5 (not duplicated here).

Table B.1.3a: Baseline-Adjusted LF/HF Ratio Correlations with NASA-TLX

NASA-TLX Dimension	β simulation	SE sim	95% CI simulation	p simulation	β baseline	SE base	95% CI baseline	p baseline
Overall Workload	+0.089	0.134	[-0.173, 0.352]	0.505	-0.053	0.133	[-0.313, 0.207]	0.690
Mental Demand	+0.054	0.111	[-0.165, 0.272]	0.629	-0.034	0.115	[-0.259, 0.191]	0.769
Physical Demand	+0.143	0.133	[-0.118, 0.403]	0.283	-0.054	0.128	[-0.304, 0.197]	0.674
Temporal Demand	+0.121	0.095	[-0.065, 0.307]	0.201	-0.035	0.099	[-0.229, 0.159]	0.727
Effort	+0.035	0.130	[-0.220, 0.290]	0.786	-0.015	0.123	[-0.256, 0.226]	0.904
Frustration	+0.039	0.146	[-0.246, 0.325]	0.788	-0.070	0.140	[-0.345, 0.204]	0.616
Performance	+0.021	0.134	[-0.242, 0.283]	0.877	-0.054	0.132	[-0.312, 0.204]	0.682

Table B.1.3b: Baseline-Adjusted Heart Rate Correlations with NASA-TLX

NASA-TLX Dimension	β simulation	SE sim	95% CI simulation	p simulation	β baseline	SE base	95% CI baseline	p baseline
Overall Workload	+0.227	0.179	[-0.124, 0.578]	0.205	-0.324	0.201	[-0.717, 0.070]	0.107

Mental Demand	+0.091	0.159	[-0.220, 0.402]	0.565	-0.172	0.178	[-0.521, 0.177]	0.335
Physical Demand	+0.058	0.181	[-0.296, 0.412]	0.747	-0.088	0.200	[-0.481, 0.305]	0.662
Temporal Demand	+0.249	0.133	[-0.012, 0.510]	0.061	-0.264	0.153	[-0.564, 0.036]	0.084
Effort	-0.122	0.170	[-0.456, 0.212]	0.473	+0.018	0.188	[-0.351, 0.387]	0.924
Frustration	+0.337	0.186	[-0.028, 0.701]	0.070	**-0.487**	0.205	**[-0.888, -0.086]**	**0.017**
Performance	+0.287	0.181	[-0.068, 0.643]	0.113	-0.299	0.201	[-0.693, 0.095]	0.137

$p < 0.05$

B.2 Subgroup Analysis

Sample: N=62-68 subjects across five subgroup analyses

Statistical Models:

- HRV Reactivity: `HRV ~ Condition × Group + Study + (1|Subject)`

- NASA-TLX: `NASA_TLX ~ Group + Study + (1|Subject)` for categorical; `NASA_TLX ~ Continuous_Variable + Study + (1|Subject)` for continuous

- Motion Metrics: `Motion ~ Group + Study + (1|Subject)` for categorical; `Motion ~ Continuous_Variable + Study + (1|Subject)` for continuous

B.2.1 Prior CCAT Training Effects

Comparison: Trained (N=30) vs Untrained (N=38).

Table B.2.1a: HRV Reactivity by Prior CCAT Training Status

Model: `Outcome ~ Condition × Training + Study + (1|Subject)`

Sample: Trained N=31, Untrained N=37.

Metric	Interaction β	95% CI	p-value	Cohen's d
RMSSD (ms)	-9.16	[-19.59, 1.27]	0.085	-0.32
LF/HF Ratio	0.44	[-0.93, 1.80]	0.530	0.13
**Heart Rate (bpm)**	**8.96**	**[3.36, 14.56]**	**0.002**	**0.44**

Interaction β represents differential baseline-to-simulation change for Trained vs Untrained

$p < 0.01$

Table B.2.1b: NASA-TLX Ratings by Prior CCAT Training Status

Model: `NASA_TLX ~ Training + Study + (1|Subject)`

Sample: Untrained N=40 scenarios, Trained N=49 scenarios.

Dimension	No Training Median [IQR]	Yes Training Median [IQR]	β	95% CI	p-value	Cohen's d
Overall Workload	36.55	41.18	4.24	[-3.58, 12.06]	0.288	0.25
Mental Demand	41.18	57.50	8.79	[-2.70, 20.28]	0.134	0.36
Physical Demand	16.18	20.00	8.83	[-0.96, 18.63]	0.077	0.43
Temporal Demand	38.24	57.50	9.61	[-3.37, 22.59]	0.147	0.34
Performance	30.88	37.50	-1.14	[-11.72, 9.44]	0.833	-0.05
Effort	47.06	50.00	1.47	[-10.04, 12.97]	0.803	0.06
Frustration	23.53	17.50	-1.80	[-14.12, 10.53]	0.775	-0.07

β represents difference for Trained vs Untrained

Table B.2.1c: Motion Metrics by Prior CCAT Training Status

Model: `Motion ~ Training + Study + (1|Subject)`

Sample: Untrained N=40 scenarios, Trained N=49 scenarios.

Metric	No Training Median	Yes Training Median	β	95% CI	p-value	Cohen's d
Acc SD (m/s^2)	0.142	0.150	0.010	[-0.009, 0.029]	0.290	0.28
Acc Max (m/s^2)	0.908	0.910	0.020	[-0.035, 0.074]	0.474	0.19
Speed Mean (km/h)	0.155	0.178	0.005	[-0.041, 0.051]	0.831	0.06
Speed SD (km/h)	0.610	0.563	0.006	[-0.100, 0.112]	0.912	0.03
Speed Max (km/h)	3.440	3.360	0.028	[-0.269, 0.326]	0.851	0.05
Distance (m)	5.200	4.700	0.267	[-1.075, 1.608]	0.697	0.10
Cadence Active Time (%)	6.458	6.667	0.441	[-1.036, 1.917]	0.559	0.15

β represents difference for Trained vs Untrained

B.2.2 Job Role Effects

Comparison: Respiratory Therapists (RT, N=21) vs Physicians (MD, N=26) vs Nurses (RN, N=21).

Note: Pairwise comparisons conducted: RT vs MD, RT vs RN, MD vs RN.

Table B.2.2a: HRV Reactivity by Job Role - Pairwise Interactions

Model: `Outcome ~ Condition × Role + Study + (1|Subject)` (tested pairwise)

Sample: RN N=32, MD N=19, RT N=17.

Metric	Comparison	Interaction β	95% CI	p-value	Cohen's d
RMSSD (ms)	RN vs MD	10.03	[-2.83, 22.89]	0.126	0.32
RMSSD (ms)	RN vs RT	-2.94	[-17.28, 11.40]	0.688	-0.09
**RMSSD (ms)**	**MD vs RT**	**-12.69**	**[-23.06, -2.32]**	**0.017**	**-0.63**
LF/HF Ratio	RN vs MD	-1.20	[-2.82, 0.43]	0.150	-0.33
LF/HF Ratio	RN vs RT	-0.46	[-2.09, 1.17]	0.581	-0.16
LF/HF Ratio	MD vs RT	0.69	[-1.15, 2.53]	0.463	0.19
**Heart Rate (bpm)**	**RN vs MD**	**-6.79**	**[-12.41, -1.17]**	**0.018**	**-0.35**
Heart Rate (bpm)	RN vs RT	6.82	[-0.57, 14.22]	0.071	0.32
**Heart Rate (bpm)**	**MD vs RT**	**13.74**	**[5.54, 21.94]**	**0.001**	**0.67**

Interaction β represents differential baseline-to-simulation change between job roles

p < 0.05, *p < 0.01*

Table B.2.2b: NASA-TLX Ratings by Job Role

Model: `NASA_TLX ~ Role + Study + (1|Subject)` (tested pairwise)

Sample: RN N=40 scenarios, MD N=26 scenarios, RT N=23 scenarios.

Dimension	RN Median [IQR]	MD Median [IQR]	RT Median [IQR]	RN vs MD β (p-value)	RN vs RT β (p-value)	MD vs RT β (p-value)
Overall Workload	39.25 [26.10, 51.06]	33.58 [25.73, 53.69]	40.20 [35.23, 46.04]	1.59 (0.740)	—	—
Mental Demand	48.53 [20.44, 60.00]	47.06 [26.73, 77.24]	47.06 [40.00, 66.25]	5.13 (0.455)	—	—
**Physical Demand**	**17.65 [5.88, 20.59]**	20.30 [6.62, 38.12]	**20.00 [14.71, 39.12]**	8.67 (0.094)	**10.41 (0.041***)*	p>0.22
Temporal Demand	52.94 [28.31, 76.73]	41.18 [20.59, 73.75]	52.50 [32.35, 78.75]	-3.41 (0.653)	—	—
Performance	35.29 [22.02, 56.25]	27.94 [17.65, 45.59]	37.50 [21.77, 59.41]	-4.51 (0.421)	—	—
Effort	42.65 [29.85, 68.24]	52.94 [29.56, 73.12]	50.00 [39.12, 55.88]	5.82 (0.392)	—	—
Frustration	20.59 [10.29, 50.73]	19.12 [6.29, 37.50]	23.53 [10.88, 40.59]	-1.89 (0.782)	—	—

p < 0.05

Table B.2.2c: Motion Metrics by Job Role

Model: `Motion ~ Role + Study + (1|Subject)` (pairwise comparisons)

Sample: RN N=40 scenarios, MD N=26 scenarios, RT N=23 scenarios.

Metric	RN Median [IQR]	MD Median [IQR]	RT Median [IQR]	RN vs MD β (p-value)	RN vs RT β (p-value)	MD vs RT β (p-value)
Acc SD (m/s^2)	0.141 [0.119, 0.161]	0.145 [0.131, 0.169]	0.158 [0.133, 0.175]	-0.001 (0.924)	—	—
Acc Max (m/s^2)	0.910 [0.858, 0.964]	0.905 [0.860, 0.980]	0.900 [0.875, 0.943]	-0.031 (0.333)	—	—
Speed Mean (km/h)	0.153 [0.082, 0.208]	0.179 [0.123, 0.252]	0.183 [0.125, 0.240]	0.002 (0.937)	—	—
Speed SD (km/h)	0.544 [0.407, 0.687]	0.586 [0.516, 0.776]	0.631 [0.474, 0.758]	-0.003 (0.965)	—	—
Speed Max (km/h)	3.329 [2.939, 3.792]	3.395 [3.042, 3.873]	3.444 [2.985, 3.677]	-0.113 (0.523)	—	—
Distance (m)	4.050 [2.225, 6.050]	5.075 [3.550, 7.200]	5.900 [3.750, 7.775]	-0.204 (0.787)	—	—
**Cadence Active Time (%)**	**5.833 [3.958, 7.813]**	6.875 [5.208, 8.594]	**8.333 [6.667, 10.000]**	0.500 (0.530)	**1.93 (0.024***)*	p>0.14

p < 0.05

B.2.3 Sex Differences

Comparison: Male (N=35) vs Female (N=34).

Table B.2.3a: HRV Reactivity by Sex

Model: `Outcome ~ Condition × Sex + Study + (1|Subject)`

Sample: Males N=35, Females N=34.

Metric	Interaction β	95% CI	p-value	Cohen's d
RMSSD (ms)	-9.67	[-20.31, 0.97]	0.075	-0.34
LF/HF Ratio	-0.19	[-1.56, 1.17]	0.781	-0.06
Heart Rate (bpm)	-5.11	[-10.97, 0.75]	0.087	-0.25

Interaction β represents differential baseline-to-simulation change for Males vs Females

Negative β for RMSSD/HR indicates Males show larger decreases/increases

Table B.2.3b: NASA-TLX Ratings by Sex

Model: `NASA_TLX ~ Sex + Study + (1|Subject)`

Sample: Females N=42 scenarios, Males N=47 scenarios.

Dimension	Female Median	Male Median	β	95% CI	p-value	Cohen's d
Overall Workload	38.86	39.22	1.15	[-6.06, 8.36]	0.754	0.07
Mental Demand	42.65	52.94	4.73	[-5.92, 15.39]	0.384	0.20
Physical Demand	20.00	17.65	-1.70	[-10.65, 7.26]	0.710	-0.08
Temporal Demand	51.25	52.50	4.31	[-7.72, 16.34]	0.483	0.15
Performance	40.00	29.41	-4.25	[-13.78, 5.28]	0.382	-0.19
Effort	41.18	52.94	9.11	[-1.18, 19.40]	0.083	0.39
Frustration	26.47	20.59	-6.20	[-17.31, 4.90]	0.273	-0.24

β represents difference for Males vs Females

Table B.2.3c: Motion Metrics by Sex

Model: `Motion ~ Sex + Study + (1|Subject)`

Sample: Females N=42 scenarios, Males N=47 scenarios.

Metric	Female Median	Male Median	β	95% CI	p-value	Cohen's d
Acc SD (m/s²)	0.149	0.145	-0.005	[-0.022, 0.012]	0.561	-0.14
Acc Max (m/s²)	0.903	0.910	-0.010	[-0.060, 0.040]	0.698	-0.10
Speed Mean (km/h)	0.168	0.167	-0.020	[-0.063, 0.022]	0.344	-0.23
Speed SD (km/h)	0.612	0.556	-0.058	[-0.154, 0.039]	0.240	-0.29
**Speed Max (km/h)**	**3.455**	**3.300**	**-0.281**	**[-0.546, -0.016]**	**0.038**	**-0.48**
Distance (m)	5.100	4.600	-0.816	[-2.032, 0.400]	0.189	-0.31
Cadence Active Time (%)	6.875	6.250	-0.515	[-1.863, 0.833]	0.454	-0.18

β represents difference for Males vs Females (negative values = higher in Females)

$p < 0.05$

B.2.4 Age Effects (Continuous)

Analysis: Age as continuous predictor.

Sample: N=68-71 subjects, age range 24-55 years (median=33 years).

Table B.2.4a: HRV Reactivity by Age

Model: `Outcome ~ Condition × Age_continuous + Study + (1|Subject)`

Sample: N=68 subjects.

Metric	Interaction β (per year)	95% CI	p-value	Cohen's d
RMSSD (ms)	0.246	[-0.473, 0.966]	0.502	0.009
LF/HF Ratio	-0.077	[-0.168, 0.014]	0.096	-0.023
Heart Rate (bpm)	-0.098	[-0.500, 0.304]	0.633	-0.005

Interaction β represents change in baseline-to-simulation reactivity per year of age

Table B.2.4b: NASA-TLX Ratings by Age

Model: `NASA_TLX ~ Age + Study + (1|Subject)`

Sample: N=89 observations from 71 subjects.

Dimension	β (per year)	95% CI	p-value	Cohen's d
Overall Workload	0.172	[-0.313, 0.658]	0.487	0.079
Mental Demand	0.479	[-0.237, 1.194]	0.190	0.154
Physical Demand	0.282	[-0.321, 0.884]	0.359	0.107
Temporal Demand	0.156	[-0.660, 0.971]	0.708	0.043
Performance	-0.273	[-0.915, 0.368]	0.404	-0.096
Effort	0.308	[-0.397, 1.012]	0.392	0.102
Frustration	0.018	[-0.738, 0.773]	0.963	0.005

β represents change in NASA-TLX rating per year of age

Table B.2.4c: Motion Metrics by Age

Model: `Motion ~ Age + Study + (1|Subject)`

Sample: N=160 observations from 71 subjects.

Metric	β (per year)	95% CI	p-value	Cohen's d
Acc SD (m/s^2)	-0.00002	[-0.0015, 0.0015]	0.983	-0.002
Acc Max (m/s^2)	0.0006	[-0.0083, 0.0096]	0.889	0.011
Speed Mean (km/h)	-0.00002	[-0.0022, 0.0022]	0.985	-0.001
Speed SD (km/h)	-0.0007	[-0.0073, 0.0058]	0.827	-0.018
Speed Max (km/h)	-0.0042	[-0.0380, 0.0296]	0.806	-0.020
Distance (m)	-0.013	[-0.079, 0.053]	0.701	-0.031
Cadence Active Time (%)	-0.010	[-0.093, 0.073]	0.809	-0.020

β represents change in motion metric per year of age

B.2.5 Years of Practice Effects (Continuous)

Analysis: Years of practice as continuous predictor

Sample: N=66-68 subjects, experience range 1-23 years (median=4 years).

Table B.2.5a: HRV Reactivity by Years of Practice

Model: `Outcome ~ Condition × YrsPractice_continuous + Study + (1|Subject)`

Sample: N=66 subjects.

Metric	Interaction β (per year)	95% CI	p-value	Cohen's d
RMSSD (ms)	0.295	[-0.752, 1.343]	0.581	0.010
LF/HF Ratio	-0.060	[-0.191, 0.072]	0.375	-0.018
Heart Rate (bpm)	-0.079	[-0.655, 0.498]	0.789	-0.004

Interaction β represents change in baseline-to-simulation reactivity per year of practice

Table B.2.5b: NASA-TLX Ratings by Years of Practice

Model: `NASA_TLX ~ YrsPractice + Study + (1|Subject)`

Sample: N=86 observations from 68 subjects.

Dimension	β (per year)	95% CI	p-value	Cohen's d
Overall Workload	0.187	[-0.540, 0.914]	0.614	0.065
Mental Demand	0.641	[-0.435, 1.716]	0.243	0.156
Physical Demand	0.789	[-0.086, 1.664]	0.077	0.225
Temporal Demand	0.436	[-0.788, 1.660]	0.485	0.091
Performance	-0.913	[-1.846, 0.019]	0.055	-0.240
Effort	0.342	[-0.698, 1.383]	0.519	0.087
Frustration	-0.246	[-1.371, 0.879]	0.668	-0.056

β represents change in NASA-TLX rating per year of practice

Table B.2.5c: Motion Metrics by Years of Practice

Model: `Motion ~ YrsPractice + Study + (1|Subject)`

Sample: N=154 observations from 68 subjects.

Metric	β (per year)	95% CI	p-value	Cohen's d
Acc SD (m/s²)	0.0007	[-0.0015, 0.0029]	0.536	0.052
Acc Max (m/s²)	0.0010	[-0.0119, 0.0139]	0.877	0.013
Speed Mean (km/h)	0.0022	[-0.0009, 0.0053]	0.169	0.117
Speed SD (km/h)	0.0025	[-0.0068, 0.0118]	0.594	0.046
Speed Max (km/h)	-0.0013	[-0.0496, 0.0470]	0.958	-0.004
Distance (m)	0.042	[-0.051, 0.135]	0.377	0.076
Cadence Active Time (%)	0.045	[-0.075, 0.165]	0.461	0.064

β represents change in motion metric per year of practice
