# Supplementary material for: Heart Rate Variability and Body Motion as Digital Biomarkers of Task Workload During Military En Route Critical Care Simulations
Source: Sensors (Basel). 2026 Jun 5;26(11):3596. doi: 10.3390/s26113596 (PMC13259423; doi:10.3390/s26113596)
Supplement: Supplementary file 1 [file sensors-26-03596-s001.zip › Supplementary materials file S3.pdf]

Supplementary materials file S3

Posture-stratified HRV sensitivity analyses

C.1 Methods

This appendix reports a posture-stratified sensitivity analysis conducted on the Experiment 2 data. The 30-minute Experiment 2 scenarios contained a scripted seated phase (the 3-minute simulated take-off, scenario minutes 7:00–10:00) embedded within an otherwise predominantly standing simulation, enabling four phase-level contrasts in which posture and operational task vary systematically.

Signal-processing and quality-control parameters (R-R range filter 300–2000 ms, ≤10% outlier removal, 20% successive-difference artifact threshold, ≥100 beats for LF/HF and ≥50 beats for RMSSD) were identical to those described in the main-manuscript Methods. The procedures specific to this analysis are described below.

C.1.1 Phase definitions

All phases were defined relative to the scenario start time (Scenario_2A_Start or Scenario_2B_Start) in each Experiment 2 subject's recording. Each subject contributed one Baseline observation and up to two observations of each scenario phase (one per Scenario 2A and 2B).

Table C.1.1: Phase definitions (All phases from Experiment 2)

Phase	Time window	Duration	Posture	Operational task
**Baseline Seated**	Baseline_Start → Baseline_End	~5 min	Seated	Resting, pre-simulation
**Standing Pre-Takeoff**	Scenario 0:00 → 7:00	7 min	Standing	Pre-flight stabilization, hands-on care
**Seated Take-off**	Scenario 7:00 → 10:00	3 min	Seated (restrained)	Care planning, team discussion, anticipation — no hands-on care
**Standing Active Care**	Scenario 10:00 → 30:00	20 min	Standing	Active in-flight care at simulated altitude

C.1.2 Native-phase HRV computation

For each subject–scenario–phase combination, RMSSD, LF/HF ratio, and mean heart rate were computed natively over the entire duration of the phase from the raw R-R interval data using NeuroKit2 (v0.2.7). This differs from the sliding-window approach used in the main pipeline (120-sec windows with 60-sec overlap aggregated by median): the phase-level approach uses all R-R intervals within a single contiguous window per phase, which is preferable for short, well-defined phases such as the 3-minute seated take-off because it avoids sliding-window overlap and boundary effects.

For each phase, a 1000 Hz peak signal was constructed from cumulative R-R times; RMSSD was obtained from `nk.hrv_time()`, LF/HF ratio from `nk.hrv_frequency()` (Welch's periodogram), and mean HR was computed as 60 / mean(R-R). One HRV value was produced per (Subject × Scenario × Phase).

C.1.3 Statistical models and contrasts

Four pre-specified contrasts were tested. Each contrast was fitted using a linear mixed-effects model with a subject random intercept:

```
Outcome ~ Phase_dummy + (1 | Subject_ID)                                [Unadjusted]
Outcome ~ Phase_dummy + Age + Sex + CCAT_training + (1 | Subject_ID)    [Adjusted]
```

Models were fitted using `statsmodels.formula.api.mixedlm` with a progressive optimizer fall-back chain (REML with `lbfgs` → ML with `lbfgs` → REML with Nelder-Mead) to ensure convergence across all metric × contrast combinations. Cohen's *d* was computed as β divided by the total within-subject standard deviation (√(residual variance + random-intercept variance)).

Table C.1.3: Pre-specified contrasts

Contrast	Phase A (reference)	Phase B	Posture	Operational task
**C1**	Baseline Seated	Seated Take-off	Matched (both seated)	Rest → cognitive engagement
**C2**	Standing Pre-Takeoff	Seated Take-off	Switch (stand → sit)	Hands-on care → seated planning
**C3**	Standing Pre-Takeoff	Standing Active Care	Matched (both standing)	Active care → active care
**C4**	Baseline Seated	Standing Active Care	Switch (sit → stand)	Rest → active simulation

C.2 Results

C.2.1 Sample

After quality-control filtering, the analysis included N=30 Experiment 2 subjects contributing 28 Baseline observations, 48 Standing Pre-Takeoff observations, 46 Seated Take-off observations, and 46 Standing Active Care observations (two subjects whose Baseline phase did not pass quality control are excluded from Baseline-involving contrasts).

C.2.2 Descriptive statistics by phase

Table C.2.2: HRV metrics by posture phase (median [Q1, Q3])

Phase	n	RMSSD (ms)	LF/HF Ratio	Heart Rate (bpm)
**Baseline Seated**	28	55.12 [41.82, 74.29]	2.63 [1.37, 4.39]	75.61 [70.97, 87.46]
**Standing Pre-Takeoff**	48	22.31 [15.57, 52.74]	4.14 [1.69, 8.10]	110.03 [97.65, 125.27]
**Seated Take-off**	46	23.45 [14.24, 53.65]	5.02 [2.66, 6.67]	96.30 [89.91, 110.95]
**Standing Active Care**	46	32.77 [22.43, 43.08]	3.89 [1.54, 7.66]	108.87 [97.12, 121.05]

C.2.3 Linear mixed-effects model contrasts

Table C.2.3a: C1 — Baseline Seated → Seated Take-off

Metric	Model	β	95% CI	p	Cohen's d	n obs	n subj*
**RMSSD (ms)**	Unadjusted	−22.86	[−35.37, −10.34]	<0.001	−0.80	74	28
	Adjusted	−23.15	[−35.95, −10.35]	<0.001	−0.79	74	28
**LF/HF Ratio**	Unadjusted	+1.81	[+0.37, +3.26]	0.014	+0.57	74	28
	Adjusted	+1.79	[+0.34, +3.25]	0.015	+0.57	74	28
**Heart Rate (bpm)**	Unadjusted	+20.24	[+16.35, +24.14]	<0.001	+1.43	74	28
	Adjusted	+20.24	[+16.33, +24.14]	<0.001	+1.45	74	28

\n subj for Baseline-involving contrasts reflects the 28 subjects with paired observations in both phases. The linear mixed-effects model included all 30 Experiment 2 subjects via the subject random intercept; the contrast estimate (Phase_dummy β) is identified by the 28 subjects providing both Baseline and Seated Take-off observations.*

Table C.2.3b: C2 — Standing Pre-Takeoff → Seated Take-off

Metric	Model	β	95% CI	p	Cohen's d	n obs	n subj
**RMSSD (ms)**	Unadjusted	+1.31	[−7.37, +9.99]	0.767	+0.04	94	30
	Adjusted	+1.39	[−7.29, +10.07]	0.753	+0.04	94	30
**LF/HF Ratio**	Unadjusted	+0.18	[−1.04, +1.41]	0.768	+0.05	94	30
	Adjusted	+0.18	[−1.06, +1.41]	0.780	+0.05	94	30
**Heart Rate (bpm)**	Unadjusted	−10.98	[−13.54, −8.41]	<0.001	−0.66	94	30
	Adjusted	−10.97	[−13.53, −8.40]	<0.001	−0.70	94	30

Table C.2.3c: C3 — Standing Pre-Takeoff → Standing Active Care

Metric	Model	β	95% CI	p	Cohen's d	n obs	n subj
**RMSSD (ms)**	Unadjusted	+4.83	[−1.32, +10.97]	0.124	+0.17	94	30
	Adjusted	+4.88	[−1.27, +11.01]	0.120	+0.17	94	30
**LF/HF Ratio**	Unadjusted	−0.05	[−1.33, +1.24]	0.944	−0.01	94	30
	Adjusted	−0.05	[−1.33, +1.22]	0.934	−0.01	94	30
**Heart Rate (bpm)**	Unadjusted	−1.16	[−3.46, +1.15]	0.326	−0.07	94	30
	Adjusted	−1.15	[−3.45, +1.16]	0.329	−0.07	94	30

Table C.2.3d: C4 — Baseline Seated → Standing Active Care

Metric	Model	β	95% CI	p	Cohen's d	n obs	n subj*
**RMSSD (ms)**	Unadjusted	−19.19	[−31.31, −7.07]	0.002	−0.75	74	28
	Adjusted	−20.67	[−32.40, −8.94]	<0.001	−0.80	74	28
**LF/HF Ratio**	Unadjusted	+1.62	[−0.12, +3.36]	0.068	+0.42	74	28
	Adjusted	+1.72	[−0.02, +3.47]	0.053	+0.45	74	28
**Heart Rate (bpm)**	Unadjusted	+30.37	[+26.27, +34.48]	<0.001	+1.95	74	28
	Adjusted	+30.44	[+26.32, +34.55]	<0.001	+2.03	74	28

\n subj for Baseline-involving contrasts reflects the 28 subjects with paired observations in both phases (see footnote to Table C.2.3a).*
